# Different Transcriptional Control of Metabolism and Extracellular Matrix in Visceral and Subcutaneous Fat of Obese and Rimonabant Treated Mice

**DOI:** 10.1371/journal.pone.0003385

**Published:** 2008-10-13

**Authors:** Carine Poussin, Diana Hall, Kaori Minehira, Anne-Marie Galzin, David Tarussio, Bernard Thorens

**Affiliations:** 1 Center for Integrative Genomics and Department of Physiology, University of Lausanne, Lausanna, Switzerland; 2 Sanofi-Aventis, Rueil Malmaison, France; University of Bremen, Germany

## Abstract

**Background:**

The visceral (VAT) and subcutaneous (SCAT) adipose tissues play different roles in physiology and obesity. The molecular mechanisms underlying their expansion in obesity and following body weight reduction are poorly defined.

**Methodology:**

C57Bl/6 mice fed a high fat diet (HFD) for 6 months developed low, medium, or high body weight as compared to normal chow fed mice. Mice from each groups were then treated with the cannabinoid receptor 1 antagonist rimonabant or vehicle for 24 days to normalize their body weight. Transcriptomic data for visceral and subcutaneous adipose tissues from each group of mice were obtained and analyzed to identify: i) genes regulated by HFD irrespective of body weight, ii) genes whose expression correlated with body weight, iii) the biological processes activated in each tissue using gene set enrichment analysis (GSEA), iv) the transcriptional programs affected by rimonabant.

**Principal Findings:**

In VAT, “metabolic” genes encoding enzymes for lipid and steroid biosynthesis and glucose catabolism were down-regulated irrespective of body weight whereas “structure” genes controlling cell architecture and tissue remodeling had expression levels correlated with body weight. In SCAT, the identified “metabolic” and “structure” genes were mostly different from those identified in VAT and were regulated irrespective of body weight. GSEA indicated active adipogenesis in both tissues but a more prominent involvement of tissue stroma in VAT than in SCAT. Rimonabant treatment normalized most gene expression but further reduced oxidative phosphorylation gene expression in SCAT but not in VAT.

**Conclusion:**

VAT and SCAT show strikingly different gene expression programs in response to high fat diet and rimonabant treatment. Our results may lead to identification of therapeutic targets acting on specific fat depots to control obesity.

## Introduction

Obesity is characterized by an increase in white fat mass, which results from an excess in food intake relative to energy expenditure. It is often associated with insulin resistance, dyslipidemia and hypertension, a cluster of conditions referred to as the metabolic syndrome, which is a major risk factor for the development of type 2 diabetes and cardiovascular diseases.

The increase in adipose tissue mass results from fat cells enlargement due to increased lipid storage, but also from recruitment and differentiation of adipocyte precursors [1]–[3]. Expansion of adipose tissue is associated with a remodeling of the extracellular matrix (ECM) and angiogenesis [4], events triggered by the production, by adipocytes, of ECM proteins and remodeling proteases such as members of the matrix metalloproteinase (MMPs), their inhibitors (TIMPs) [5]–[7], or cathepsins [8], [9] and the production of angiogenic factors such as VEGF or leptin [10]–[12].

Storage of fat in adipose tissue is limited and exceeding this capacity leads to accumulation of lipids in other tissues, in particular muscle, liver, and the endocrine pancreas, and to the secretion by adipocytes of various adipokines. Ectopic fat accumulation and adipokines then combine to worsen insulin resistance and to induce beta-cell secretory dysfunctions [13]–[15] [16]. Remarkably, increasing the capacity of fat tissue to store lipids in ob/ob mice by transgenic overexpression of adiponectin leads to massive obesity but improved metabolic control secondary to reduced ectopic fat deposition [17]. Improvement of the metabolic syndrome can also be achieved by reducing adiposity. For instance, pharmacological treatment with the cannabinoid receptor 1 (CB1) antagonist rimonabant induces weight loss in obese rodents [18] and human [19], [20]. This effect is mediated by a modulation of the hypothalamic melanocortin pathway, which increases energy expenditure [21], [22] but possibly also through the regulation of adipocyte differentiation and function [23], [24]. Thus, the control of total fat mass and the mechanisms limiting fat tissue expansion are intimately linked in the control of metabolic disease progression.

The adipose tissue consists, however, of several depots, located at different anatomical sites [25], which may originate from distinct precursors [26], [27], and which have different physiological functions and pathophysiological roles [28]. The visceral, as opposed to the subcutaneous adipose depots, may contribute more to the defects associated with the metabolic syndrome [29], [30]. This may result from a more rapid turnover of lipids [31] [32] and to the direct venous drainage from the visceral fat to the liver [33]. On the other hand, a certain amount of subcutaneous fat appears to be beneficial for longevity and health in ageing people whereas visceral fat is detrimental [34].

Mouse models of diet-induced obesity are considered to be relevant to the study of human obesity. In previous studies, we showed that genetically homogenous C57Bl/6 mice fed a HFD develop various degrees of glucose intolerance and obesity. In large cohorts, approximately 50% of the mice become obese and diabetic, ∼15% remain lean but glucose intolerant, ∼15% remain lean with a normal glucose tolerance, and the rest of the mice have intermediate phenotypes [35]. This differential metabolic adaptation to the same feeding conditions may be caused by yet uncharacterized epigenetic modifications [36], [37].

Here, we fed C57Bl/6 mice a high fat diet for 6 months and mice with different body weights but similar levels of glucose intolerance were then treated with vehicle or rimonabant for one month to normalize body weight. We then performed transcriptomic analysis of the visceral (VAT) and subcutaneous (SCAT) adipose tissues from each mouse group. This experimental design allowed us to identify genes regulated by HFD irrespective of body weight gain, genes whose expression was associated with increased body weight, and genes that were normalized by rimonabant treatment. Together our data show strikingly different adaptation of both VAT and SCAT to high fat diet, suggesting that largely different tissue remodeling events take place for expansion of both fat depots and their normalization by rimonabant treatment. Our data may provide new targets for regulating selectively visceral or subcutaneous fat development in obesity.

## Results

### Physiological characterization of HFD fed mice treated or not with rimonabant

**Figure 1:**
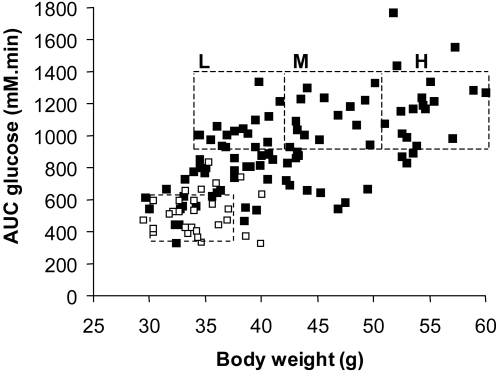
Effect of high fat diet on body weight and glucose intolerance. Mice were fed for 6 months with a normal chow (open squares) or a high fat diet (closed squares) and their body weight and tolerance to intraperitoneal glucose injection (AUC glucose) were assessed. The figure shows the heterogenous adaptation of C57Bl/6 mice to the HFD. Groups of mice (in squares) with low (L), medium (M), or high (H) final body weight but with similar glucose intolerance were selected for subsequent analysis.

**Figure 2:**
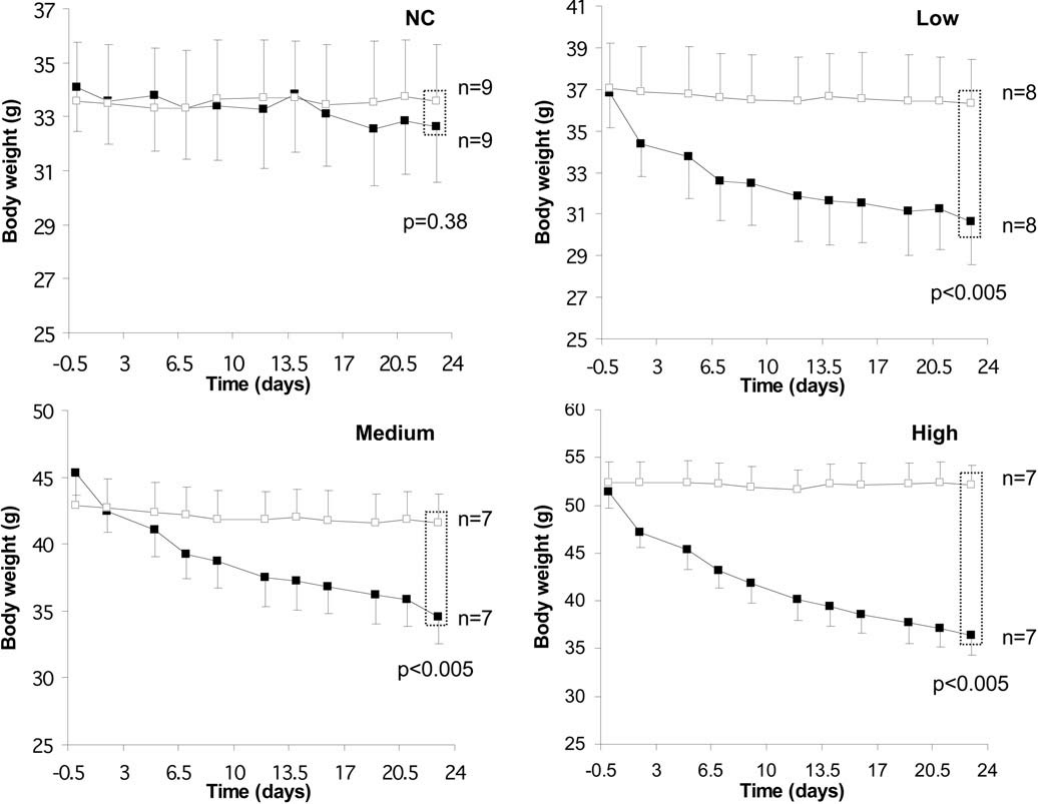
Effect of rimonabant on body weight. After 6 months of diet, mice under NC or HFD mice of the L, M and H groups were treated with vehicle (open squares) or rimonabant (closed squares) for one month while maintained on the same diet and. The body weight of each mouse was monitored every 2–3 days during this treatment. Results are expressed as mean±SD, n = 6. p-values indicate statistical significance for the comparison of body weight between vehicle and rimonabant treatments using *t*-test following a test of variance homogeneity (F-test).

**Figure 3:**
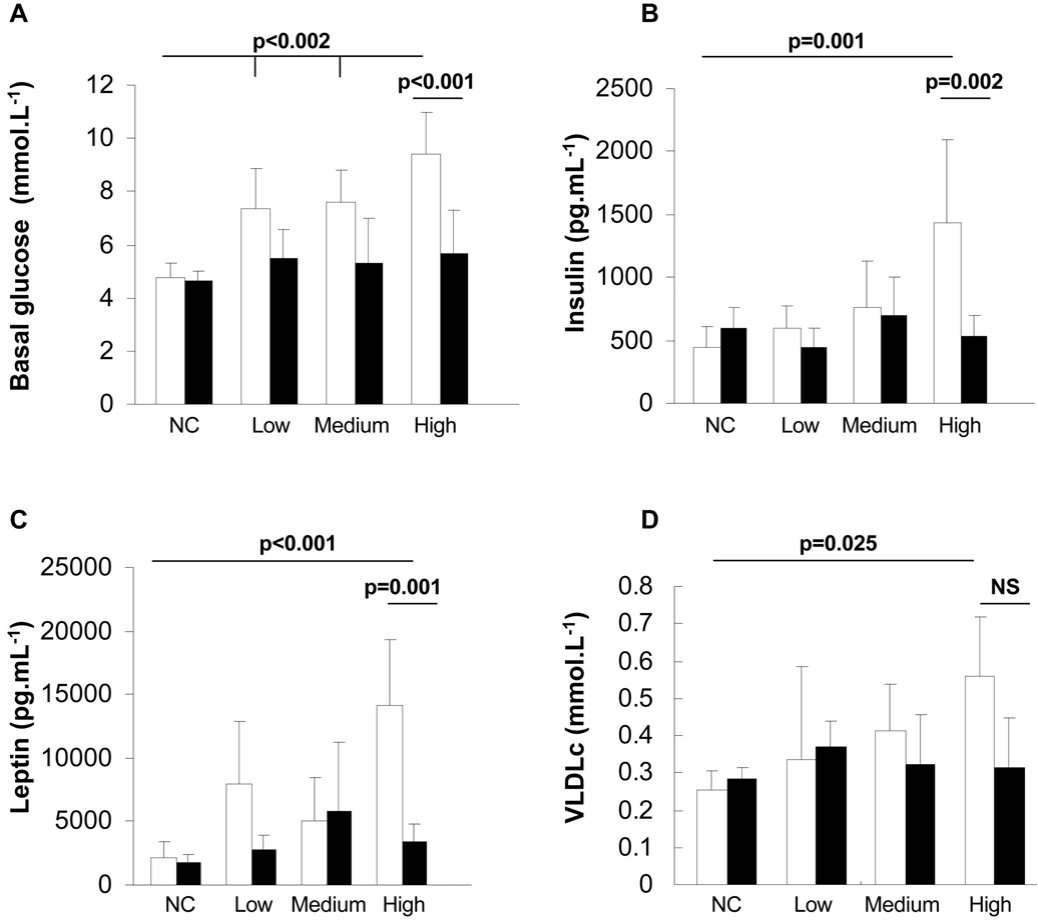
Effect of rimonabant on glycemia and insulin, leptin and VLDL plasma concentrations. At the end of the 1-month rimonabant or vehicle treatment, glycemia (A), plasma insulin (B), leptin (C) and VLDL (D) were measured in the fasted state in the normal chow (NC) or in the HFD fed mice of each subgroup. Results are expressed as mean±SD (number of mice/measurement: 3–9).

**Figure 4:**
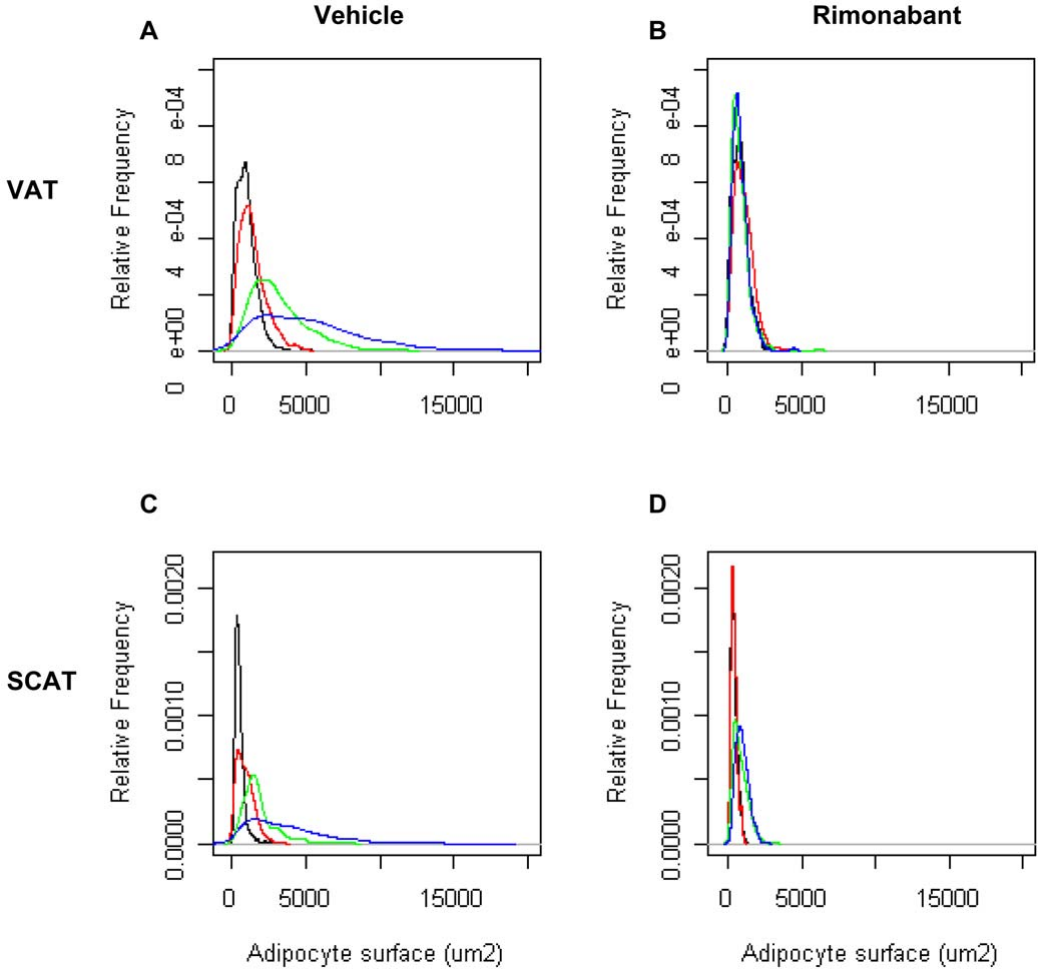
Effect of HFD and rimonabant treatment on adipocyte cross sectional area in VAT and SCAT. The figure shows the average cross-sectional area of adipocytes from the L (red), M (green) and H (blue) groups and from the NC fed mice (black), as measured on adipose tissue sections (≥420 adipocytes measured per tissue). A, B: adipocytes from VAT after vehicle or rimonabant treatment, respectively. C, D, adipocytes from SCAT after vehicle or rimonabant treatment, respectively. Increased body weight correlated with increased adipocytes cross-sectional areas. rimonabant treatment restored normal size distribution.

Mice were fed a normal chow or a high fat diet for 6 months and their body weight and tolerance to an intraperitoneal glucose injection were measured. These parameters showed that the adaptation of each mouse to the HFD was very heterogeneous (Figure 1), as previously described [35]. For the subsequent transcriptomic study, we selected mice (boxes in Figure 1) that were similarly glucose intolerant but with low (L), medium (M) or high (H) body weight. The normal chow-fed mice (NC, open squares in Figure 1) were used as a reference. At six months of HFD or NC feeding mice in each group were randomly distributed into two subgroups, and received either a daily oral administration of rimonabant or vehicle for 24 days, while remaining on either the HFD or NC diet. As shown in Figure 2, this led to a remarkable correction of body weight in each of the HFD mouse group but no change in body weight in NC mice. The body weight of the mice from each HFD group treated with rimonabant was not different no longer different from that of the NC group at the end of the treatment. In the H group this was associated with a correction of basal glycemia and insulinemia (Figure 3A, B), a significant reduction in leptin levels and a tendency to normalize plasma VLDL (Figure 3C, D). Adipocyte cross-sectional area in the VAT and SCAT was also assessed in each group of mice (Figure 4A,C). There was a direct correlation between body weight and mean cross-sectional area in both tissues, suggesting that adipocyte hypertrophy is an important contributor of fat tissue expansion. This hypertrophy was corrected by rimonabant treatment in all groups (Figure 4B,D).

### Analysis of genes regulated by HFD

#### Microarray experiments and statistical analysis

**Figure 5:**
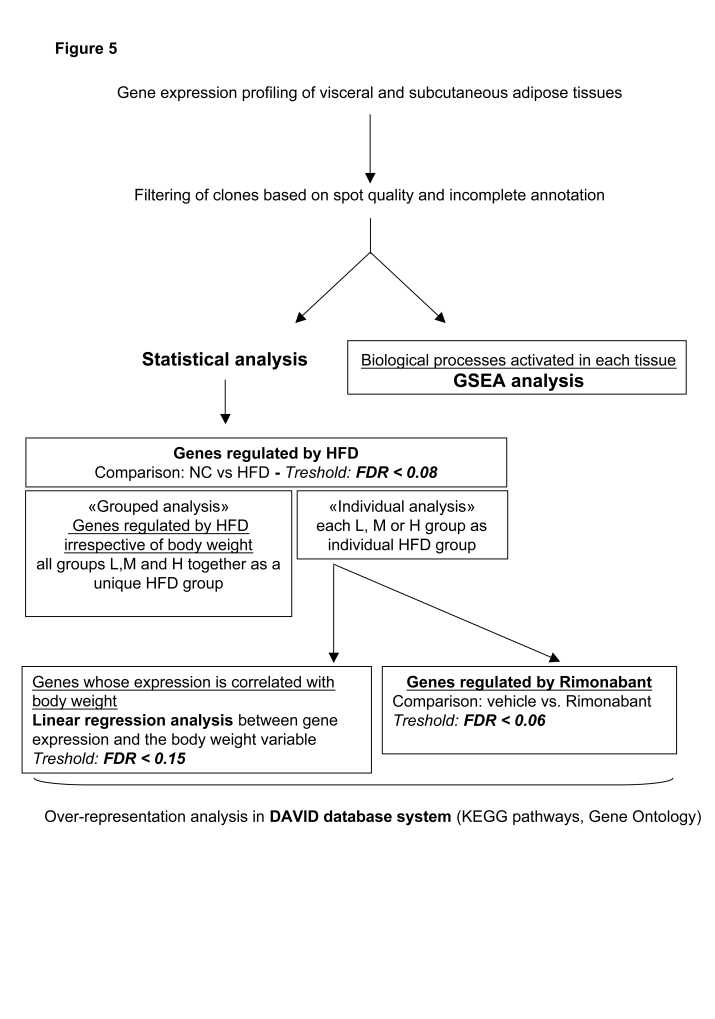
Data analysis workflow. Normalized microarray data were filtered based on spot quality and incomplete annotation and used for Gene Set Enrichment Analysis (GSEA). Alternatively, statistical analysis was performed to reveal the effect of HFD feeding on gene expression irrespective of body weight, i.e., considering the mice from the L, M, and H group as a single group (“grouped analysis”). Separately, genes specifically regulated in the L, M, and H groups as compared to the NC group were identified to determine, by linear regression analysis, those whose expression level correlated with body weight (”individual analysis”). Genes regulated by rimonabant were selected by comparing their expression level separately in the L, M or H mice treated or not with rimonabant.

Total RNA was prepared from visceral and subcutanous adipose tissues from the L, M, H and the NC mouse groups, treated with vehicle or rimonabant. Transcript profiling was performed using microarrays containing 17'664 mouse cDNAs. The filtered data for each group of HFD mice were then analyzed as shown in Figure 5. Geneset enrichment analysis (GSEA) was conducted on the filtered data. Separately, statistical analysis was performed to identify the effect of HFD on gene expression. This analysis was performed, firstly, to search for genes whose expression was regulated irrespective of body weight, comparing the NC group with the L, M and H mice considered as a unique HFD group (“Grouped analysis”). Secondly, we identified genes that were significantly regulated by HFD in each of the L, M or H groups (“Individual analysis”) and searched, by linear regression analysis those whose expression level was correlated with body weight. The genes that were affected by rimonabant treatment were also identified from these gene sets. The pathways over-represented in each gene set were analyzed by KEGG analysis and Gene Ontology using the David database system.

**Figure 6:**
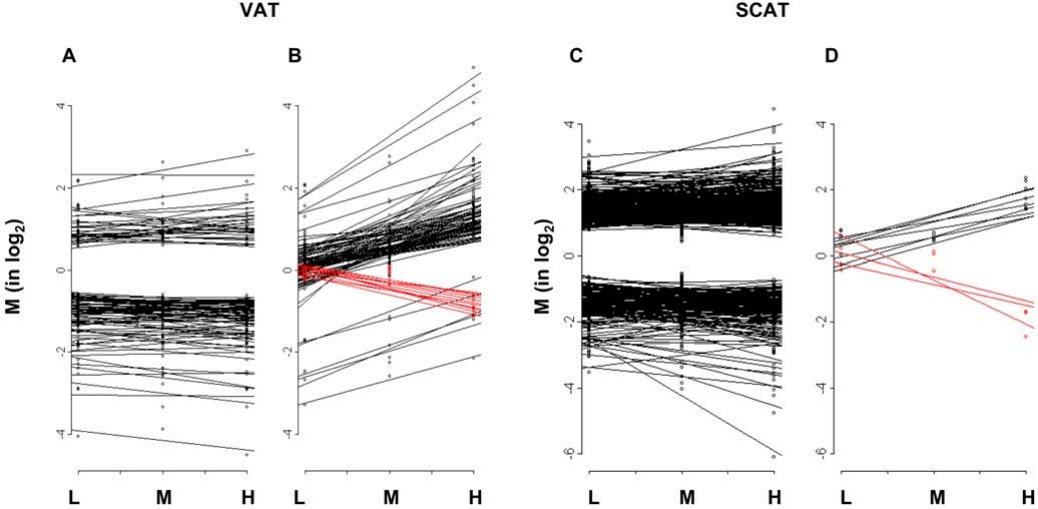
High fat diet feeding induces differential regulation of gene expression in VAT and SCAT. The figure illustrates the numbers of genes that are similarly regulated in the L, M and H groups, irrespective of body weight gain (A, C) or whose expression is positively or negatively correlated with body weight (B, D) in the VAT (A,B) and SCAT (C,D). The two tissues behaved very differently, with the VAT having a larger number of genes whose expression was correlated with body weight than SCAT. SCAT, however, had a much greater number of genes regulated by HFD but most were similarly regulated irrespective of body weight. Data are expressed as the mean of M values calculated for each gene in each group (n = 4–5 mice). Red lines represent genes, which were negatively correlated with body weight.

Figure 6 shows a global representation of the number of genes, with their expression level (M value), that were up- or down-regulated irrespective of body weight gain in VAT and SCAT (Fig 6A,C) and those whose expression was regulated in proportion to body weight gain in both tissues (Fig 6B,D). The figure shows that both tissues responded differently to HFD. There were many more genes that were either up- or down-regulated by HFD irrespective of body weight in the SCAT than in the VAT. On the other hand, there was a much larger proportion of genes which had their expression level correlated with body weight in the VAT than in the SCAT. These gene expression data are now described separately for each type of analysis.

#### Genes regulated by high fat diet irrespective of body weight gain

In the VAT, there were 41 genes that were up-regulated and 85 that were down-regulated by HFD feeding, irrespective of body weight gain (Figure 6A). Among the up-regulated genes there was a significant over-representation of genes involved in “cytoskeleton/cell projection biogenesis” (Table 1, and Table S1 for the identity of individual genes). Among the down-regulated genes, there was a significant enrichment in genes pertaining to the glycolytic pathway, and to the fatty acid and steroid biosynthesis pathways (Table 1, and Table S1) suggesting similar inhibition by HFD of glycolysis, lipogenesis and steroid biosynthesis in all mouse subgroups.

**Table 1:** Biological functions enriched in the groups of genes that were similarly regulated by HFD in the L, M and H in VAT or in SCAT (“Grouped analysis”).

Tissue	Regulation	Biological function		P-value
***VAT***	UP	**Cytoskeleton/Cell projection biogenesis (motility)**		*2.9E-02/6.9E-03*
	DOWN	**Carbohydrate metabolism**		*7.6E-12*
			Glycolysis	1.8E-10
			Glycogen metabolism	1.5E-03
			Pyruvate metabolism	5.3E-05
		**Lipid metabolism**		*2.3E-07*
			Steroid metabolism (cholesterol biosynthesis)	1.3E-05
			Fatty acid metabolism	2.7E-04
***SCAT***	UP	**Cytoskeleton/Adhesion**	Cell junction	3.2E-03
			Cytoskeleton	2.1E-03
		**Metabolism**	Lipid metabolism	5.0E-02
			Fatty acid metabolism	2.4E-06
			Coenzyme metabolism	6.5E-03
		**Intracellular signaling**	Small GTPase	1.9E-03
			dephosphorylation	2.6E-02
		**Transport**	Intracellular transport protein	3.9E-03
		**Ubiquitin pathway**	Ubl conjugation pathway	3.2E-02
	DOWN	**Immune response**		1.5E-03
		**Ribosome**		3.5E-04
		**Transport**	Glucose transport	5.8E-03

In SCAT, 342 genes were up-regulated and 175 were down-regulated similarly in all three subgroups of HFD mice (Figure 6C). The up-regulated genes contained genes that belonged to the “cytoskeleton/adhesion”, “metabolism”, “intracellular signaling”, “transport” and “ubiquitination” biological functions (Table 1, and Table S2). Among the down-regulated genes there was enrichment in genes pertaining to the “immune response”, “ribosome” and “transport” categories.

The above data indicated very striking difference of adaptation of both tissues to HFD with different classes of functions being modulated in each tissue. Alternatively, some metabolic pathways were regulated in the same direction but through regulation of different genes. For instance, “glycolysis” was down-regulated in VAT, as suggested by the reduced expression of 9 genes, including hexokinase, phosphofructokinase, pyruvate kinase and pyruvate dehydrogenase (Table 1 and Table S1), whereas in SCAT, the “glycolysis” class of function was not significantly regulated. However, the three genes that were down-regulated in the “transport” class of function were *Glut1*, *Glut4* and *Glut8*, suggesting decreased capacity for glucose utilization in SCAT through a decreased uptake capacity. Similarly, the “fatty acid metabolism” genes that were up-regulated in SCAT contained 10 genes encoding β-oxidation enzymes, suggesting increased fatty acid oxidation capacity (Table 1 and Table S1), whereas only *Cpt1* (carnitine palmitoyltransferase 1) and *Acadm* (Acetyl-Coenzyme A dehydrogenase, medium chain) genes were increased in VAT (not shown).

**Genes regulated by high fat diet in proportion to body weight gain**

We next searched among the genes that were significantly regulated by HFD feeding in the L, M and H groups those whose expression level was correlated with body weight.

**Figure 7:**
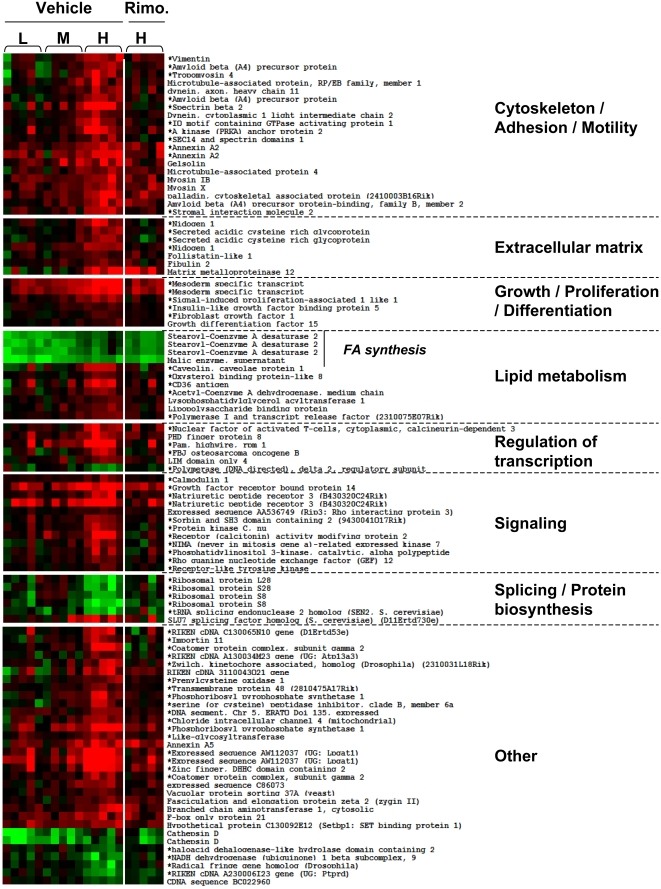
Gene whose expression was correlated with body weight in VAT and their correction by rimonabant treatment. The genes that were selected by linear regression analysis for positive or negative correlation with body weight were classified according to biological function categories and their expression level was displayed for each mouse with red: increased expression, green: decreased expression as compared to NC fed mice. * indicates genes whose expression was significantly regulated by rimonabant in the H group.

In VAT, linear regression analysis identified 77 genes (89 probesets) that were positively correlated with body weight and 10 (11 probesets), which were negatively correlated (Figure 6B). Figure 7 shows the expression pattern of these genes in each group and their classification in specific biological functions. Strikingly, a large proportion of the genes positively correlated with body weight (23 genes) encoded proteins pertaining to the “cytoskeleton/adhesion/motility and extracellular matrix” biological functions and thus involved in maintaining cell and tissue structure. Other genes encoded proteins involved in “growth/proliferation/differentiation” (5 genes), “regulation of transcription” (6 genes), “signaling” (11 genes) and “splicing/protein biosynthesis” (5 genes). Only 8 metabolic genes had their expression level correlated with body weight: stearoyl-Coenzyme A desaturase 2 (*Scd2*) and malic enzyme, which are involved in lipogenesis, had markedly reduced expression levels in the L group and close to normal expression in the H group as compared to the NC control; acetylCoA dehydrogenase, medium chain (*Acadm*), lysophosphatidyl glycerol acyl transferase (*Lpgat1*), caveolin1 (*Cav1*), CD36, oxysterol-binding protein-like 8 (*Osbpl8*), lipopolysaccharide binding protein (*Lbp*), polymerase I and transcript release factor (*Ptrf*) were positively correlated with body weight.

**Figure 8:**
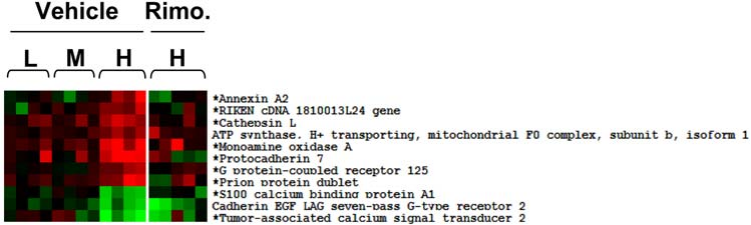
Gene whose expression was correlated with body weight in SCAT and their correction by rimonabant treatment. The genes that were selected by linear regression analysis for positive or negative correlation with body weight are displayed for each mouse with red: increased expression, green: decreased expression as compared to NC fed mice. * indicate genes whose expression was significantly regulated by rimonabant in the H group.

In SCAT, only 8 genes were positively and 3 negatively correlated with body weight (Figure 6D and Figure 8). They belonged to cell and tissue structure and remodeling class of function (annexin A2, cathepsin L, protocadherin7, cadherin EGF LAG seven pass G-type receptor 2), and to various function but not to “metabolism”.

Together the above data suggest that in VAT, two different transcriptional programs were induced by HFD feeding. One controls metabolic adaptation by down-regulating lipid and steroid biosynthesis and glucose catabolism, and by increasing free fatty acid beta-oxidation. This program is regulated irrespective of body weight and adipocytes size. The second one, constituted by genes whose expression was correlated with body weight, controls the adipocyte cytoarchitecture and extracellular matrix. The increased expression of genes implicated in differentiation, growth and proliferation, regulation of transcription, splicing and biosynthesis, also suggests that recruitment and differentiation of adipocyte precursors contributes to fat mass expansion. In contrast, in SCAT, the “metabolism” and “structure” genes were mostly regulated irrespective of body weight. These two fat depots therefore respond to the HFD with very different transcriptional programs, even though they show similar adipocyte hypertrophy with increasing body weight.

### GSEA analysis

As a separate bioinformatics analysis, GSEA was performed to identify genesets enriched in VAT and SCAT of mice from the H compared to the NC group (Table 2 and Table S3).

**Table 2:** Best gene sets obtained by GSEA for the comparison of NC and H groups treated with saline (NC vs. H) or Rimonabant (NCT vs. HT) in VAT and in SCAT.

Tissue	Comparison	Regulation of genes	Gene set name	Number of matched genes from gene set	Number of leading edge genes	Normalized enrichment score (NES)	p-value
**VAT**	**NC vs. H**	up in H	ST_p38_MAPK_Pathway	23	12	2.22	0.000
		up in H	ST_JNK_MAPK_Pathway	23	13	1.94	0.004
		up in H	ST_Differentiation_Pathway_in_PC12_Cells	24	15	1.77	0.013
		up in H	CR_CAM (Cancer-related genes involved in cell adhesion and metalloproteinases)	42	25	1.8	0.014
		up in H	EMT_UP (Epithelial-to-mesenchymal transition)	33	13	1.78	0.018
		up in H	SFT (Solitary Fibrous Tumor)	65	33	1.51	0.054
	**NCT vs. HT**	up in HT	mRNA splicing	29	23	1.87	0.009
**SCAT**	**NC vs. H**	up in H	MAP00561_Glycerolipid_metabolism	23	10	1.85	0.006
		up in H	RAR_UP (Genes upregulated by retinoic acid receptor)	22	12	1.76	0.014
		up in H	ST_JNK_MAPK_Pathway	20	15	1.51	0.045
		up in H	ST_Differentiation_Pathway_in_PC12_Cells	22	19	1.58	0.053
	**NCT vs. HT**	up in HT	GLUCOSE_DOWN	81	51	1.81	0.006
		down in HT	VOXPHOS	48	39	−2.02	0.019
		down in HT	Electron_Transport_Chain	51	38	−1.93	0.033

In both adipose tissues of HFD fed mice, there was an enrichment in genes belonging to the p38 and JNK MAP kinase pathways (ST_p38_MAPK_Pathway, in VAT and ST_JNK_MAPK_Pathway, in VAT and SCAT), which are known to be involved in adipogenesis [38], [39], and a specific gene set related to cell differentiation (ST_Differentiation_Pathway_in_PC12_ Cells) in both tissues. Interestingly, the leading edge genes for the JNK pathway were largely different between the two adipose tissues, with 13 leading edge genes in the VAT and 15 in the SCAT and only eight common genes (Table S3). For the PC-12 Differentiation set there were 15 leading edge genes in VAT and 19 in SCAT and 13 were common.

Genesets associated with regulation of cytoskeleton and extracellullar matrix (CR_CAM) as well as migration/motility (EMT_UP) processes were enriched only in VAT of the H mice, which also showed an interesting enrichment in the SFT geneset. This geneset is enriched in genes for the extracellular matrix and some remodelling enzymes and is considered as a signature of activated tissue stroma [40] (Table 2 and Table S3). This further suggested that the expansion of the adipose tissue was associated with cell proliferation and rearrangement of the cytoskeleton, the extracellular matrix and the participation of stromal cells.

In SCAT, GSEA identified an enrichment in genes controlling glycerolipid metabolism (MAP00561_Glycerolipid_metabolism) and genes regulated by retinoic acid (RAR_UP).

Together the above data indicated that very different mechanisms operate during fat mass expansion in VAT and SCAT. In particular, there was a differential activation of the MAP kinase pathway and, in VAT, a more extensive remodeling of cell structure and extracellular matrix and an important participation of its stromal environment.

### Rimonabant effect on VAT and SCAT gene expression

Rimonabant treatment normalized body weight (Figure 2) and adipocyte cross-sectional area in both VAT and SCAT (Figure 4B, D). To analyze the effect of rimonabat on gene expression we focused our analysis on the obese (H) group of mice, treated with rimonabant or vehicle. In VAT from these mice, among the 595 genes that were regulated by HFD, 206 genes had their expression normalized by rimonabant treatment and these belonged to each class of biological function (Table S4). Among the genes whose expression was correlated with body weight (Figure 7), 64% (56 out of 87) had their expression normalized by rimonabant treatment. They also fell into each class of function. In the groups of genes that were similarly regulated in the three HFD groups, only 6% (7 out of 115) had their expression normalized by rimonabant (Table S1). Three belonged to the glycolysis pathway: *Glut4*, phosphomannomutase, pyruvate carboxylase; the other were fatty acid synthase, a sialyltransferase (*Siat10*), a DEAD box-containing protein (*Ddx27*) and a Riken clone (4930504E06).

In the SCAT from obese H mice, among the 698 genes that were regulated by HFD, 186 genes had their expression normalized by rimonabant treatment. Among the genes whose expression was correlated with body weight (Figure 8), 81% (9 out of 11) had their expression normalized by rimonabant treatment. In the group of genes that were similarly regulated in the three HFD groups, 17% (87 of the 517) had their expression normalized by rimonabant treatment (Table S2).

We next performed GSEA on gene expression data from the adipose tissues of mice from the HFD (H group) and NC group treated with rimonabant. Two major observations were made. Firstly, in both VAT and SCAT, the genesets that were detected by GSEA in comparing the adipose tissues of HFD vs. NC fed mice were no longer detected. Secondly, new gensets were identified in each adipose tissue. In VAT, there was an up-regulation of a “mRNA splicing” gene set, whereas, interestingly, in SCAT there was an up-regulation of glucose-sensitive genes (GLUCOSE_DOWN) and, intriguingly, a down-regulation of two sets of genes encoding oxidative phosphorylation proteins (VOXPHOS and Electron_Transport_Chain) (Table 2). As presented in Table S3, there was a coordinated decreased expression of a total of 35, 14, 3, 6, and 9 genes encoding subunits of, respectively, complexes I to V. Thus, GSEA indicated a near-normalization of the transcripts expressed in each tissue after rimonabant treatment, with however, indication that changes in mRNA splicing accompanied normalization of VAT structure and function whereas SCAT normalization was associated with an increased sensitivity to glucose and decreased oxidative phosphorylation.

## Discussion

Here, we used C57Bl/6 mice fed a high fat diet, and which develop different degrees of obesity, to analyze the differential adaptation of visceral and subcutaneous fat to HFD feeding and the mechanisms associated with rimonabant-induced body weight normalization. Bioinformatic analysis of our transcriptomic data revealed very different behaviors of both adipose tissues. These differences indicated different transcriptional control of genes involved in nutrients metabolism, in the control of adipocytes precursor recruitment and differentiation, as well as in the control of adipose cytoarchitecture and extracellular matrix. Correction of body weight and adipocytes size by rimonabant treatment normalized the expression of most genes in both tissues with, however, a striking coordinated down-regulation of oxidative phosphorylation genes in SCAT and up-regulation of mRNA splicing activity in VAT.

### Genes regulated by HFD and correlated or not with body weight

In VAT, genes regulated by HFD in the L, M and H groups, showed similar down-regulation of a large set of genes coding for enzymes of glycolysis, lipogenesis and steroid biosynthesis and up-regulation of β-oxidation genes, *Cpt1a* and *Acadm*. Linear regression analysis of gene expression and body weight revealed a large number of genes encoding proteins of the extracellular matrix and the cytoskeleton (Figure 6). Thus, the differential fat tissue expansion observed in C57Bl/6 mice under HFD was associated with up-regulation of a large group of genes required for reorganization of the interaction between the adipocyte cytostructure and its tissue environment. Fat tissue expansion was also associated with increased expression of several genes involved in lipid transport, uptake and storage, and which have previously been correlated with lipid accumulation and fat cell enlargment, *Cav1*, *CD36*, *Mest*, *Fgf1*, *Gdf15* [37], [41]–[43].

In SCAT, as compared to VAT, many more genes were up- or down-regulated similarly in all three mouse subgroups and only 8 genes were correlated with body weight. Seventeen “structure” genes were similarly regulated in the three subgroups by HFD and three had expression levels positively correlated with body weight (annexin2, protocadherin 7, cathepsin L) whereas the seven-pass cadherin-EGF protein (*Celsr2*), involved in homophilic cell-cell interactions, showed negative correlation with body weight. Thus, as reported in several previous studies [7], [37], [44], [45], remodeling of the interaction between the adipocyte cytoskeleton, integrins, and extracellular matrix proteins is a key event in fat cell expansion, which is also associated with angiogenesis required for adipose tissue development. Our data, however, indicate that this tissue remodeling process proceeds differently in both subcutaneous and visceral fat. In particular, whereas there is a striking correlation between fat cell enlargement and expression of these “structure” genes in VAT, this is not the case in the SCAT where most of these genes are increased even when adipocytes are small. The basis for this differential response is not known but may be related to both tissues originating from different types of precursor cells, having different differentiation potential [26], [27].

Another interesting difference between both tissues is in the regulated expression of “metabolic” genes. For instance, gene expression data suggest that glycolysis was decreased in both tissues. However, in the VAT this was associated with a down-regulation of several genes encoding glycolytic enzymes, whereas in SCAT there was only a reduction in the glucose transporters *Glut1*, *Glut4* and *Glut8*. Similarly, fatty acid synthesis appeared to be reduced in both tissues as a consequence of reduced expression of 8 fatty acid synthesis genes in VAT and only 2 in SCAT, with *Fasn* being reduced in both tissues. For the fatty acid β-oxidation only *Cpt1a* and *Acadm* was increased in VAT and 10 in SCAT (including *Cpt1a*). Thus, adipocytes from both tissues appear to modulate major nutrient metabolism pathways in the same direction but using different gene expression responses.

### Gene set enrichment analysis of VAT and SCAT

GSEA provided complementary information about the mechanisms of VAT and SCAT adaptation to high fat diet in the H as compared to the NC group. Firstly, there was a significant enrichment in genes implicated in the JNK MAP kinase pathway in both tissues and of the p38 pathway only in VAT. The MAP kinase pathways are known to be involved in adipogenesis but their exact contribution is still partially controversial [38]. For instance, the p38 MAP kinase has been proposed to activate adipogenesis [46], [47] whereas more recent evidence indicates that it rather inhibits adipogenesis [48]. The JNK pathway is activated in adipose tissue of obese animals and JNK^−/−^ mice are resistant to diet-induced obesity [39], thus this pathway may be required for adipose tissue growth in obesity, consistent with our observation that the JNK pathway is activated in both tissues. Strikingly however, only approximately half of the genes in the leading edge of the JNK pathway geneset were common to VAT and SCAT, further supporting the existence of various molecular mechanisms to control the same biological process.

Secondly, GSEA showed an enrichment in VAT, but not in SCAT, of genes from the “EMT -UP” geneset. This contains genes expressed during embryonic development and in pathological epithelial-to-mesenchymal differentiation associated with malignant tumor progression and metastasis [49], [50]. GSEA also identified the SFT gene set in the VAT of the obese (H) mice. This gene set was originally derived from the study of different fibroblastic tumors, solitary fibrous tumors (SFT) and desmoid-type fibromatosis (DFT) [40]. Both tumors are derived from fibroblasts and consist of cells with similar morphology but different behavior, the DFT tumors being more aggressive. These fibroblastic tumors were analyzed by micorarray analysis and genesets were identified that distinguished the two forms of tumors. The SFT gene set was enriched in genes for the extracellular matrix and some remodelling enzyme whereas the DTF gene set was enriched for genes characteristic of a fibrotic response and with activation of the TGF and Wnt signaling pathways. These signatures therefore provide evidence that the tissue stroma may follow different patterns of differentiation involving distinct activation of extracellular matrix proteins, remodeling enzymes and growth and differentiation response. The presence of the SFT geneset in the VAT but not the SCAT therefore indicates that expansion of the visceral fat is associated with a specific pattern of activation of the tissue stroma. As a recent report suggests that the SFT geneset may also contain a signature for the presence of mesenchymal stem cells [51]. This further suggests that the process of adipogenesis and precursor recruitment may be different between these two tissues. Thus our statistical analysis provides indication that expansion of the visceral and subcutaneuous tissues in obesity is not only directed by adipocyte-specific responses but also by the close interaction between the adipocytes and their stromal environment. This may contribute to the specific function of the differentiated adipocytes in each fat depot.

### Effect of rimonabant treatment

Rimonabant treatment normalized the body weight of mice under HFD and improved basal glycemia, and plasma insulin, leptin and VLDL concentrations, in line with previous reports [52], [53]. Statistical analysis of gene expression indicated that expression of a large proportion of the “structure” and many of the “lipid metabolism” genes were normalized in VAT and SCAT of obese mice. GSEA of genes expressed in VAT or SCAT of the H and NC mice after rimonabant treatment indicated normalization of gene expression. In particular, the geneset related to proliferation, differentiation, and tissue remodeling/plasticity processes were no longer detected (Table 2), which suggests that rimonabant reduces adipose tissue differentiation, in agreement with a previous report [24]. In VAT there was an increased in the “mRNA splicing” geneset upon rimonabant treatment. This set is enriched in several genes encoding regulators of gene splicing (Table S3). Thus, expansion of adipose tissue during obesity or its normalization by rimonabant treatment may require not only changes in level of expression of many genes but also the production of new protein isoforms issued from differential mRNA splicing. The identity of the concerned genes is, however, not yet known.

Very interestingly also, GSEA showed a decreased expression of genes involved in oxidative phosphorylation in SCAT. This was surprising as decreased expression of oxidative phosphorylation genes in muscle and adipose tissue was reported to correlate with, and inferred to cause insulin resistance and type 2 diabetes [54]–[57]. However, in a very recent report [58], the link between reduced oxidative phosphorylation and whole body glucose metabolism was directly addressed by studying mice with tissue-specific deletion of the AIF gene, which leads to reduced expression of electron transport chain genes to a level similar to that reported in studies of type 2 diabetic patients [54], [55]. The consequence of this deregulation was, however, to confer greater insulin sensitivity and to protect the mice against diet-induced obesity. This was explained by the requirement for a greater utilization of nutrients to generate a lower amount of ATP when the oxidative phosphorylation capacity was decreased. It is thus very striking that in the SCAT a very large number of oxidative phosphorylation genes were deregulated by rimonabant treatment. Thus, rimonabant may control body weight, in part at least, by down-regulating oxidative phosphorylation genes, an event that is specific to SCAT.

We did not detect changes in oxidative phosphorylation genes expression in the VAT of HFD or HFD and rimonabant treated mice. This is in contrast with a recent report showing that oxidative phosphorylation genes were down-regulated by HFD and increased by rimonabant treatment [59]. However, this study was performed with epididymal fat whereas we studied omental fat and the different observations may be related to the different visceral fat depots analyzed, as well as to differences in high fat diet composition. It is also worth noting,that rimonabant had no significant effect on gene regulation in the adipose tissue of NC fed mice.

### Concluding remarks

It is important to note that we studied whole adipose tissue depots and not isolated adipocytes. Thus, our data provide information on the adaptation of each adipose tissue to HFD feeding and it is not possible to strictly define which genes are expressed specifically in adipocytes as compared to those expressed in various stromal cells including mesenchymal stem cell, preadipocytes, fibroblasts, inflammatory or endothelial cells. However, our statistical analysis of the microarray data, offers a unique way at looking at the specific adaptation of both VAT and SCAT to HFD and their normalization by CB1 antagonism.

We observed, in VAT, that “metabolic” genes were similarly regulated independently of body weight whereas the “structure” genes had expression levels correlated with increased body weight and fat cell size. This indicated a need for reorganization of cytoarchitecture, extracellular matrix and for the activation of the tissue stroma during fat mass expansion. In the SCAT a larger number of genes were up- or down-regulated by HFD than in VAT, but most of these genes were regulated irrespective of body weight gain and GSEA suggested a less active adipogenesis and no major involvement of the tissue stroma. Blockade of CB1 receptor activity suggested a striking decreased in oxidative phosphorylation in SCAT and a changing pattern of mRNA splicing in VAT. An interesting challenge of future research will be to analyze the basis for the differential response of these two tissues, in particular, whether this is due to differences between the interaction of (pre)adipocytes with their stromal environment or to adipocytes originating from distinct precursors with different capacity to respond to the high fat diet, or a combination of both. In addition, our data may help identify new targets for the selective regulation of the function and expansion of either the subcutaneous or visceral fat in obesity.

## Materials and Methods

### Animals and treatments

Six-week-old C57BL/6J male mice (Janvier, France) were fed a normal chow (NC; 12% fat, 28% protein, and 60% carbohydrate) or a high fat diet (HFD; 72% fat, 28% protein, and <1% carbohydrate) for 7 months. Their body weight and tolerance to intraperitoneal glucose injections (1 g/kg body weight) (AUC: area under the curve) were determined after 3, 6 and 7 months of HFD as described previously [35]. After 6 months of HFD, mice with AUC values of 920–1400 mM.min were selected. These mice were divided into 3 groups based on their body weight (Figure 1). Eighteen NC mice were also selected. Each mouse was treated with vehicle (0.1% Tween 80 (Fluka) in H2O) or rimonabant (10 mg.kg^−1^.day^−1^) administrated by gavage daily for 1 month. Vehicle or rimonabant treatments were randomly assigned in each group of NC and HFD mice. The body weight of mice under treatment was monitored every 2–3 days. At the end of the treatment, omental (VAT) and subcutaneous (SCAT) adipose tissues, as well as plasmas, were collected and immediately frozen in liquid nitrogen. The animal experiments have been approved by the Service Vétérinaire Candonal of the Canton de Vaud.

### Plasma measurements

Leptin and insulin plasma concentrations were assessed by ELISA (Mouse Adipokines from LINCO) using the Luminex system (Biorad). Free fatty acids (FFA), HDL, LDL, cholesterol, triglycerides (TG) concentrations were measured enzymatically with commercial kits (FFA: n°999–75406 from Wako Pure Chemicals Industries; HDL-C plus: n°03030024, LDL-C plus: n°03038661, cholesterol: n°12016630; TG: n°12016648 from Roche Diagnostic), using a Hitachi robot 902 (Roche). Glycemia were measured from tail vein blood using a Glucometer (Glucotrend Premium; Boehringer Mannheim GmbH).

### Adipocyte area measurement

Sections (20 µm) of visceral and subcutaneous adipose tissue were cut with a cryostat at −30°C, fixed in 10% paraformaldehyde for 10 minutes and stained with hematoxilin-eosin. Cross-sectional area were measured on 420 cells/group using a Carl Zeiss AxioVision program.

### RNA preparation, labeling and hybridization on cDNA microarrays

RNA from 5 different mice per group was extracted from visceral and subcutaneous adipose tissues using peqGOLD Trifast™ (peqlab) and chloroform-isoamylalcool (24∶1) extraction. RNA was precipitated with isopropanol and purified by passage over RNeasy columns (Qiagen). RNA quality was checked before and after amplification with a Bioanalyzer 2100 (Agilent). RNA was reverse transcribed and RNA was amplified with MessageAmp™ kit (Ambion). A Mouse Universal Reference (Clontech) was similarly amplified and both adipose tissue and reference RNAs were labeled by an indirect technique with Cy5 and Cy3 according to published protocols [60]. Labeled RNAs were hybridized to microarrays containing 17664 cDNAs prepared at the DNA Array Facility of the University of Lausanne. Scanning, image, and quality control analyses were performed as previously published [60]. Data were expressed as log_2_ intensity ratios (Cy5/Cy3), normalized with a print tip locally weighted linear regression (Lowess) method and filtered based on spot quality and incomplete annotation. All analysis were performed with the R software for statistical computing available at the Comprehensive R Archive Network (cran.us.r-project.org/).

### Analysis of gene expression and statistics

The flow chart for data analysis is described in figure 5. Statistically differentially expressed genes were identified using the limma package from Bioconductor (http://www.bioconductor.org). Gene Set Enrichment Analysis (GSEA) was performed directly using filtered data from VAT or SCAT of the different mouse groups (http://www.broad.mit.edu/gsea/msigdb/genesets.jspcollectionC2) [61]. The ranking of genes was based on *t*-statistic values calculated by limma.

KEGG pathway or Gene Ontology analysis were carried out using DAVID (http://david.abcc.ncifcrf.gov)[62].

Statistical comparisons of plasma insulin, leptin and VLDL were performed using a one-way analysis of variance followed by a Tukey post-hoc test (SPSS). The statistical significance for the comparison of body weight curves obtained in the presence of vehicle or rimonabant treatment for NC or HFD subgroups was evaluated with linear models with repeated measures using SPSS program. Statistical significance for the comparison of body weight between vehicle and rimonabant treatments at day-23 was assessed by a Student's *t*-test after a variance homogeneity test (F-test).

The microarray data have been deposited in the GEO data base under ID n° GSE11790.

## Supporting Information

Table S1 (0.09 MB DOC)

Table S2 (0.17 MB DOC)

Table S3 (0.32 MB DOC)

Table S4 (0.21 MB DOC)
