# Supplementary material for: Different Transcriptional Control of Metabolism and Extracellular Matrix in Visceral and Subcutaneous Fat of Obese and Rimonabant Treated Mice
Source: PLoS One. 2008 Oct 13;3(10):e3385. doi: 10.1371/journal.pone.0003385 (PMC2586343; doi:10.1371/journal.pone.0003385)
Supplement: Table S1 — (0.09 MB DOC) [file pone.0003385.s001.doc]

**Table S1: List of genes from VAT associated with the table 1 (“Grouped analysis”).**

|  |  |  |  |  | **M values** | | | |
| --- | --- | --- | --- | --- | --- | --- | --- | --- |
|  | **Refseq / Genbank** | **Gene symbol** |  | **Gene description** | **HFD** | **L** | **M** | **H** |
| **UP** |  |  |  |  |  |  |  |  |
| **Cytoskeleton / Cell projection biogenesis (motility)** | | | | | | | | |
|  | NM_009041.1 | Rdx |  | Radixin | 1.05 | 1.08 | 1.24 | 0.82 |
|  | NM_019765.2 | Rsn |  | Restin (Reed-Steinberg cell-expressed intermediate filament-associated protein) | 0.69 | 0.70 | 0.77 | 0.59 |
|  | NM_026163.1 | Pkp2 |  | Plakophilin 2 | 1.26 | 1.07 | 0.89 | 1.82 |
|  | NM_021356.2 | Gab1 |  | RIKEN cDNA 1700019H22 gene (Gab1: growth factor receptor bound protein 2-associated protein 1) | 0.79 | 0.82 | 0.81 | 0.75 |
|  | NM_010590.2 | Jub |  | Ajuba | 0.77 | 0.58 | 0.70 | 1.02 |
|  | NM_026116.2 | Bbs2 |  | Bardet-Biedl syndrome 2 homolog (human) | 0.71 | 0.89 | 0.64 | 0.61 |
|  |  |  |  |  |  |  |  |  |
| **DOWN** | |  |  |  |  |  |  |  |
| **Carbohydrate metabolism** | | | | | | | | |
|  | **Glycolysis** | | | | | | | |
|  | NM_013820.1 | Hk2 |  | Hexokinase 2 | -2.04 | -1.84 | -2.51 | -1.77 |
|  | NM_001001303.1;NM_008084.1 | LOC14433 |  | Glyceraldehyde-3-phosphate dehydrogenase | -1.58 | -1.49 | -1.58 | -1.68 |
|  | NM_023119.1 | Eno1 |  | Enolase 1, alpha non-neuron | -1.51 | -1.50 | -1.43 | -1.59 |
|  | NM_008826.2 | Pfkl |  | Phosphofructokinase, liver, B-type | -1.35 | -1.24 | -1.36 | -1.47 |
|  | X52379 |  |  | Enolase, a-isozym (most tissues) * | -1.18 | -1.10 | -1.11 | -1.33 |
|  | NM_011099.2 | Pkm2 |  | Pyruvate kinase, muscle | -1.14 | -1.10 | -1.31 | -1.02 |
|  | NM_007438.3 | Aldoa |  | Aldolase 1, A isoform | -1.11 | -1.26 | -1.06 | -1.00 |
|  | NM_010699.1 | Ldh1 |  | Lactate dehydrogenase 1, A chain | -0.97 | -1.03 | -1.03 | -0.86 |
|  | NM_008155.1 | Gpi1 |  | Glucose phosphate isomerase 1 | -0.92 | -0.88 | -0.79 | -1.08 |
|  | **Glycogen metabolism** | | | | | | | |
|  | NM_133198.1 | Pygl |  | Liver glycogen phosphorylase | -1.75 | -1.62 | -1.82 | -1.81 |
|  | AK028914.1 | Agl |  | Amylo-1,6-glucosidase, 4-alpha-glucanotransferase | -1.14 | -1.25 | -1.35 | -0.82 |
|  | **Pyruvate metabolism** | | | | | | | |
|  | NM_172665.1 | Pdk1 |  | Pyruvate dehydrogenase kinase, isoenzyme 1 | -1.78 | -1.96 | -1.75 | -1.64 |
|  | NM_008797.1 | Pcx | ***** | **Pyruvate carboxylase** | -1.43 | -1.51 | -0.97 | -1.81 |
|  | NM_008810.2 | Pdha1 |  | Pyruvate dehydrogenase E1 alpha 1 | -1.22 | -1.28 | -1.21 | -1.16 |
|  | NM_024221.2 | Pdhb |  | Pyruvate dehydrogenase (lipoamide) beta | -1.20 | -1.34 | -1.15 | -1.11 |
|  | **Pentose phosphate** | | | | | | | |
|  | NM_009388.2 | Tkt |  | Transketolase | -2.62 | -2.40 | -2.58 | -2.88 |
|  | AK002894 |  |  | 6-Phosphogluconate dehydrogenase | -1.26 | -1.21 | -1.42 | -1.15 |
|  | NM_009075.1 | Rpia |  | Ribose 5-phosphate isomerase A | -0.89 | -0.62 | -0.88 | -1.16 |
|  | NM_011528.1 | Taldo1 |  | Transaldolase 1 | -0.85 | -0.75 | -0.82 | -0.99 |
|  | **Fructose and mannose metabolism** | | | | | | | |
|  | NM_013872.1 | Pmm1 | ***** | **Phosphomannomutase 1** | -1.23 | -0.81 | -1.24 | -1.65 |
|  |  |  |  |  |  |  |  |  |
| **Lipid metabolism** | | | | | | | | |
|  | **Steroid metabolism (cholesterol biosynthesis)** | | | | | | | |
|  | NM_138656.1 | Mvd |  | Mevalonate (diphospho) decarboxylase | -1.97 | -1.80 | -1.93 | -2.18 |
|  | NM_026784.1 | Pmvk |  | Phosphomevalonate kinase | -1.67 | -1.71 | -1.87 | -1.45 |
|  | NM_020010.1 | Cyp51 |  | Cytochrome P450, family 51 | -1.56 | -1.79 | -1.62 | -1.28 |
|  | NM_146006.1 | Lss |  | Lanosterol synthase | -1.56 | -1.53 | -1.67 | -1.49 |
|  | NM_019657.2 | Hsd17b12 |  | Hydroxysteroid (17-beta) dehydrogenase 12 | -1.11 | -1.26 | -1.15 | -0.94 |
|  | NM_009270.2 | Sqle |  | Squalene epoxidase | -1.09 | -0.98 | -1.18 | -1.12 |
|  | NM_007856.2 | Dhcr7 |  | 7-dehydrocholesterol reductase | -0.95 | -1.03 | -0.78 | -1.05 |
|  | NM_010191.2 | Fdft1 |  | Farnesyl diphosphate farnesyl transferase 1 | -0.82 | -0.75 | -0.59 | -1.11 |
|  | **Fatty acid metabolism** | | | | | | | |
|  | NM_007988.1 | Fasn | ***** | **Fatty acid synthase** | -4.15 | -4.06 | -3.88 | -4.52 |
|  | NM_130450.1 | Elovl6 |  | ELOVL family member 6, elongation of long chain fatty acids (yeast) | -3.06 | -2.91 | -3.34 | -2.93 |
|  | NM_009381.2 | Thrsp |  | Thyroid hormone responsive SPOT14 homolog (Rattus) | -3.00 | -2.88 | -2.79 | -3.34 |
|  | NM_009127.2 | Scd1 |  | Stearoyl-Coenzyme A desaturase 1 | -2.53 | -2.54 | -2.56 | -2.48 |
|  | NM_022415.2 | Ptges |  | Prostaglandin E synthase | -1.41 | -1.28 | -1.44 | -1.52 |
|  | NM_146094.1 | Fads1 |  | Fatty acid desaturase 1 | -0.83 | -0.70 | -0.68 | -1.11 |
|  | **Lipid receptors** | | | | | | | |
|  | NM_011436.1 | Sorl1 |  | Sortilin-related receptor, LDLR class A repeats-containing | -1.92 | -1.88 | -2.10 | -1.79 |
|  | NM_010700.1 | Ldlr |  | Low density lipoprotein receptor | -1.41 | -1.37 | -1.58 | -1.28 |
|  | NM_016741.1 | Scarb1 |  | Scavenger receptor class B, member 1 | -0.97 | -1.18 | -0.76 | -0.99 |
|  | NM_013587.1 | Lrpap1 |  | Low density lipoprotein receptor-related protein associated protein 1 | -0.89 | -1.00 | -0.86 | -0.79 |

Footnotes: M values are log_2_ of the ratio of gene expression in the L, M, and H and HFD groups as compared to expression in the NC group. The HFD group represent the mean expression from the “grouped analysis” for the combined L, M and H groups. For each column, data are the mean of M values calculated with: n=15 mice for the HFD group and n=5 for L, M or H group. * indicates significantly regulated genes by rimonabant in H group.
