# Supplementary material for: Different Transcriptional Control of Metabolism and Extracellular Matrix in Visceral and Subcutaneous Fat of Obese and Rimonabant Treated Mice
Source: PLoS One. 2008 Oct 13;3(10):e3385. doi: 10.1371/journal.pone.0003385 (PMC2586343; doi:10.1371/journal.pone.0003385)
Supplement: Table S2 — (0.17 MB DOC) [file pone.0003385.s002.doc]

**Table S2: List of genes from SCAT associated with the table 1 (“Grouped analysis”).**

|  | **RefSeq / Genbank** | **Gene symbol** |  | **Gene description** | **M HFD** | **M LC** | **M MC** | **M HC** |
| --- | --- | --- | --- | --- | --- | --- | --- | --- |
| **UP** |  |  |  |  |  |  |  |  |
| **Adhesion / cytoskeleton** | | | | | | | | |
|  | **Cell junction** | | | | | | | |
|  | NM_016807.1 | Sdcbp |  | Syndecan binding protein | 2.24 | 2.52 | 1.69 | 2.53 |
|  | NM_007614.2 | Catnb |  | Catenin beta | 1.84 | 1.9 | 1.65 | 1.96 |
|  | NM_026163.1 | Pkp2 |  | Plakophilin 2 | 1.81 | 2.11 | 1.06 | 2.27 |
|  | NM_009166.1 | Sorbs1 | ***** | **Sorbin and SH3 domain containing 1** | 1.8 | 1.89 | 0.88 | 2.63 |
|  | NM_011699.2 | Lin7c |  | Lin 7 homolog c (C. elegans) | 1.48 | 1.52 | 1.36 | 1.55 |
|  | NM_009502.3 | Vcl |  | Vinculin | 1.45 | 1.3 | 1.34 | 1.71 |
|  | NM_009818.1 | Catna1 |  | Catenin alpha 1 | 1.38 | 1.46 | 1.34 | 1.32 |
|  | NM_009386.1 | Tjp1 |  | RIKEN cDNA D130061D10 gene | 1.38 | 1.2 | 1.72 | 1.21 |
|  | NM_020606.4 | Parva |  | Parvin, alpha | 1.17 | 1.29 | 0.87 | 1.37 |
|  | **Cytoskeleton** | | | | | | | |
|  | AK053672.1 | Dmxl2 |  | Dmx-like 2 | 1.94 | 2.01 | 1.64 | 2.17 |
|  | NM_009260.1 | Spnb2 | ***** | **Spectrin beta 2** | 1.64 | 1.41 | 1.34 | 2.16 |
|  | NM_028459.1 | Wasl |  | Wiskott-Aldrich syndrome-like (human) | 1.63 | 1.85 | 1.62 | 1.41 |
|  | NM_178688.2 | Ablim1 | ***** | **Actin-binding LIM protein 1** | 1.4 | 1.74 | 1.01 | 1.45 |
|  | NM_026361.1 | Pkp4 |  | Plakophilin 4 | 1.4 | 1.18 | 1.94 | 1.08 |
|  | NM_013625.1 | Pafah1b1 |  | Platelet-activating factor acetylhydrolase, isoform 1b, beta1 subunit | 1.3 | 1.3 | 1.05 | 1.55 |
|  | NM_145148.1 | Frmd4b |  | FERM domain containing 4B | 1.05 | 0.83 | 1.08 | 1.23 |
|  | NM_010578.1 | Itgb1 |  | Integrin beta 1 (fibronectin receptor beta) | 1.03 | 1.01 | 0.93 | 1.16 |
| **Metabolism** | | | | | | | | |
|  | **Lipid metabolism** | | | | | | | |
|  | NM_007616.2 | Cav1 | ***** | **Caveolin, caveolae protein 1** | 2.58 | 2.75 | 1.72 | 3.27 |
|  | NM_019760.1 | Tde2 |  | Tumor differentially expressed 2 | 2.17 | 2.06 | 1.93 | 2.52 |
|  | NM_019464.1 | Sh3glb1 | ***** | **SH3-domain GRB2-like B1 (endophilin)** | 1.53 | 1.36 | 1.29 | 1.94 |
|  | NM_030750.2 | Sgpp1 |  | Sphingosine-1-phosphate phosphatase 1 | 1.3 | 1.4 | 1.06 | 1.44 |
|  | NM_009981.2 | Pcyt1a |  | Phosphate cytidylyltransferase 1, choline, alpha isoform | 1.22 | 1.24 | 0.78 | 1.65 |
|  | NM_027976.1 |  |  | Acyl-CoA synthetase long-chain family member 5 | 1.01 | 0.92 | 0.93 | 1.18 |
|  | **Fatty acid metabolism** | | | | | | | |
|  | NM_007382.1 | Acadm | ***** | **Acetyl-Coenzyme A dehydrogenase, medium chain** | 2.64 | 2.73 | 1.65 | 3.53 |
|  | NM_013703.1 | Vldlr | ***** | **Very low density lipoprotein receptor** | 1.92 | 1.36 | 1.52 | 2.88 |
|  | NM_023733.2 | Crot | ***** | **Carnitine O-octanoyltransferase** | 1.56 | 1.47 | 1.36 | 1.86 |
|  | NM_023737.2 | Ehhadh |  | Enoyl-Coenzyme A, hydratase/3-hydroxyacyl Coenzyme A dehydrogenase | 1.46 | 1.47 | 1.45 | 1.46 |
|  | NM_008650.1 | Mut |  | Methylmalonyl-Coenzyme A mutase | 1.44 | 1.15 | 1.87 | 1.28 |
|  | NM_145558.1 | Hadhb |  | Hydroxyacyl-Coenzyme A dehydrogenase/3-ketoacyl-Coenzyme A thiolase/enoyl-Coenzyme A hydratase (trifunctional protein), beta subunit | 1.32 | 1.7 | 0.46 | 1.79 |
|  | NM_025826.1 | Acadsb |  | Acyl-Coenzyme A dehydrogenase, short/branched chain | 1.25 | 1.55 | 1.14 | 1.06 |
|  | NM_007381.2 | Acadl |  | Acetyl-Coenzyme A dehydrogenase, long-chain | 1.2 | 1.51 | 0.81 | 1.3 |
|  | NM_013495.1 | Cpt1a |  | Carnitine palmitoyltransferase 1a, liver | 1.17 | 1.2 | 1.09 | 1.22 |
|  | NM_027976.1 | Acsl5 |  | Acyl-CoA synthetase long-chain family member 5 | 1.01 | 0.92 | 0.93 | 1.18 |
|  | **Coenzyme metabolism** | | | | | | | |
|  | NM_013743.1 | Pdk4 |  | Pyruvate dehydrogenase kinase, isoenzyme 4 | 2.38 | 2.69 | 1.99 | 2.46 |
|  | NM_145962.1 | Pank3 | ***** | **Pantothenate kinase 3** | 2.19 | 2.01 | 0.74 | 3.82 |
|  | NM_145942.2 | Hmgcs1 |  | 3-hydroxy-3-methylglutaryl-Coenzyme A synthase 1 | 2.09 | 2.19 | 1.51 | 2.56 |
|  | NM_019736.2 | Acate2 |  | Acyl-Coenzyme A thioesterase 2, mitochondrial | 1.82 | 1.95 | 1.39 | 2.11 |
|  | NM_007508.2 | Atp6v1a1 | ***** | **ATPase, H+ transporting, V1 subunit A, isoform 1** | 1.72 | 1.35 | 1.31 | 2.49 |
|  | NM_133826.2 | Atp6v1h |  | ATPase, H+ transporting, lysosomal 50/57kDa, V1 subunit H | 1.69 | 2.02 | 1.12 | 1.93 |
|  | BY034856.1 |  |  | Transcribed locus | 1.56 | 1.4 | 2.04 | 1.23 |
|  | NM_011506.1 | Sucla2 |  | Succinate-Coenzyme A ligase, ADP-forming, beta subunit | 1.47 | 1.47 | 1.33 | 1.62 |
|  | NM_023281.1 | Sdha |  | Succinate dehydrogenase complex, subunit A, flavoprotein (Fp) | 1.32 | 1.54 | 1.16 | 1.25 |
|  | NM_024471.2 | Lias |  | Lipoic acid synthetase | 0.97 | 1.11 | 0.62 | 1.17 |
| **Intracellular signaling** | | | | | | | |  |
|  | **Small GTPase** | | | | | | | |
|  | NM_026697.2 | Rab14 |  | RAB14, member RAS oncogene family | 2.07 | 2.15 | 1.95 | 2.11 |
|  | NM_008112.3 | Gdi3 | ***** | **Guanosine diphosphate (GDP) dissociation inhibitor 3** | 2.06 | 1.83 | 1.91 | 2.43 |
|  | NM_017382.2 | Rab11a |  | RAB11a, member RAS oncogene family | 1.86 | 2.08 | 1.75 | 1.76 |
|  | NM_025859.1 | Arl1 | ***** | **ADP-ribosylation factor-like 1** | 1.74 | 2.06 | 0.82 | 2.34 |
|  | NM_009706.1 | Arhgap5 |  | Rho GTPase activating protein 5 | 1.61 | 1.67 | 1.64 | 1.52 |
|  | NM_026011.2 | Arl10c |  | ADP-ribosylation factor-like 10C | 1.51 | 1.51 | 1.05 | 1.96 |
|  | NM_175930.3 | Rapgef5 | ***** | **Rap guanine nucleotide exchange factor (GEF) 5** | 1.35 | 0.84 | 1.38 | 1.83 |
|  | NM_025846.1 | Rras2 | ***** | **Related RAS viral (r-ras) oncogene homolog 2** | 1.27 | 1.03 | 0.85 | 1.92 |
|  | NM_019491.4 | Rala |  | V-ral simian leukemia viral oncogene homolog A (ras related) | 1.26 | 1.36 | 1.14 | 1.3 |
|  | NM_011816.2 | E430034L04Rik |  | RIKEN cDNA E430034L04 gene | 1.17 | 1.09 | 1.13 | 1.3 |
|  | NM_008999.2 | Rab23 |  | RAB23, member RAS oncogene family | 1.01 | 0.9 | 1.11 | 1.03 |
|  | NM_009105.2 | Rsu1 |  | Ras suppressor protein 1 | 0.84 | 1.07 | 0.64 | 0.8 |
|  | **Dephosphorylation** | | | | | | | |
|  | NM_172707.1 | Ppp1cb |  | Protein phosphatase 1, catalytic subunit, beta isoform | 1.93 | 1.87 | 1.3 | 2.62 |
|  | NM_008913.1 | Ppp3ca |  | Protein phosphatase 3, catalytic subunit, alpha isoform | 1.83 | 1.89 | 1.78 | 1.82 |
|  | NM_144843.2 | Mtmr6 |  | Myotubularin related protein 6 | 1.74 | 1.71 | 1.86 | 1.65 |
|  | NM_011151.1 | Ppm1b |  | Protein phosphatase 1B, magnesium dependent, beta isoform | 1.52 | 1.92 | 1.36 | 1.28 |
|  | NM_008974.3 | Ptp4a2 | ***** | **Protein tyrosine phosphatase 4a2** | 1.5 | 1.53 | 1.3 | 1.68 |
|  | NM_008960.1 | Pten |  | Phosphatase and tensin homolog | 1.24 | 1.39 | 0.97 | 1.36 |
|  | NM_008914.1 | Ppp3cb |  | Protein phosphatase 3, catalytic subunit, beta isoform | 1.23 | 1.14 | 1.27 | 1.27 |
|  | NM_011202.2 | Ptpn11 |  | Protein tyrosine phosphatase, non-receptor type 11 | 1.22 | 1.54 | 0.78 | 1.34 |
| **Transport** | | | | | | | | |
|  | **Intracellular transport protein** | | | | | | | |
|  | NM_015827.1 | Copb2 |  | Coatomer protein complex, subunit beta 2 (beta prime) | 1.79 | 2.05 | 1.58 | 1.74 |
|  | NM_008379.2 | Kpnb1 |  | Karyopherin (importin) beta 1 | 1.36 | 1.44 | 0.98 | 1.67 |
|  | NM_010481.1 | Hspa9a |  | RIKEN cDNA D330004O07 gene | 1.51 | 1.7 | 1.26 | 1.56 |
|  | NM_148938.2 | Slc1a3 |  | RIKEN cDNA E130014H10 gene | 1.59 | 2.41 | 1.59 | 0.76 |
|  | NM_011883.1 | Rnf13 |  | Ring finger protein 13 | 1.55 | 1.56 | 1.32 | 1.76 |
|  | NM_026155.1 | Ssr3 |  | Signal sequence receptor, gamma | 1.54 | 1.51 | 1.2 | 1.91 |
|  | NM_026343.1 | Stx17 |  | Syntaxin 17 | 1.38 | 1.7 | 0.87 | 1.56 |
|  | NM_011502.1 | Stx3 |  | Syntaxin 3 | 0.98 | 0.95 | 1.1 | 0.89 |
|  | NM_028173.1 | Tram1 |  | Translocating chain-associating membrane protein 1 | 1.64 | 2.02 | 1.26 | 1.65 |
|  | NM_027016.1 | Tloc1 |  | Translocation protein 1 | 1.82 | 1.84 | 1.98 | 1.64 |
|  | NM_018753.3 | Ywhab | ***** | **Tyrosine 3-monooxygenase/tryptophan 5-monooxygenase activation protein, beta polypeptide** | 1.65 | 2.08 | 0.55 | 2.32 |
| **Ubiquitin pathway** | | | | | | | | |
|  | **Ubl conjugation pathway** | | | | | | | |
|  | NM_024213.1 | Anapc4 | ***** | **Anaphase promoting complex subunit 4** | 1.62 | 1.68 | 1.58 | 1.61 |
|  | NM_026160.1 | Map1lc3b |  | Microtubule-associated protein 1 light chain 3 beta | 1.62 | 1.74 | 1.31 | 1.82 |
|  | NM_175226.2 | Rnf139 |  | Ring finger protein 139 | 1.61 | 1.87 | 1.25 | 1.71 |
|  | NM_011543.2 | Skp1a | ***** | **S-phase kinase-associated protein 1A** | 1.47 | 1.65 | 0.83 | 1.92 |
|  | NM_025356.2 | Ube2d3 |  | Ubiquitin-conjugating enzyme E2D 3 (UBC4/5 homolog, yeast) | 1.45 | 1.71 | 0.84 | 1.79 |
|  | NM_207215.1 | Phr1 |  | Pam, highwire, rpm 1 | 1.43 | 1.24 | 1.64 | 1.4 |
|  | NM_015822.1 | Fbxl3 |  | F-box and leucine-rich repeat protein 3 | 1.43 | 1.58 | 1.27 | 1.44 |
|  | NM_010890.3 | Nedd4 |  | Neural precursor cell expressed, developmentally down-regulted gene 4 | 1.39 | 1.2 | 1.42 | 1.55 |
|  | NM_144860.2 | Mib1 | ***** | **Mindbomb homolog 1 (Drosophila)** | 1.39 | 1.26 | 1.23 | 1.7 |
|  | NM_020012.1 | Rnf14 |  | Ring finger protein 14 | 1.36 | 1.55 | 1.09 | 1.45 |
|  | NM_133758.2 | Usp47 |  | Ubiquitin specific protease 47 | 1.32 | 1.26 | 1.49 | 1.19 |
|  | NM_009481.1 | Usp9x |  | Ubiquitin specific protease 9, X chromosome | 1.2 | 0.98 | 1.4 | 1.21 |
|  | NM_019912.1 | Ube2d2 | ***** | **Ubiquitin-conjugating enzyme E2D 2** | 0.99 | 1.12 | 0.77 | 1.1 |
|  |  |  |  |  |  |  |  |  |
| **DOWN** | |  |  |  |  |  |  |  |
| **Immune response** | | | | | | | | |
|  | NM_008518.1 | Ltb |  | Lymphotoxin B | -3.63 | -2.9 | -3.2 | -4.8 |
|  | NM_013653.1 | Ccl5 |  | Chemokine (C-C motif) ligand 5 | -3.23 | -2.8 | -2.7 | -4.2 |
|  | NM_008840.1 | Pik3cd |  | Phosphatidylinositol 3-kinase catalytic delta polypeptide | -2.23 | -2.3 | -2 | -2.4 |
|  | NM_010566.1 | Inpp5d |  | Inositol polyphosphate-5-phosphatase D | -2.09 | -2.2 | -2.4 | -1.7 |
|  | NM_010386.2 | H2-DMa |  | Histocompatibility 2, class II, locus DMa | -1.77 | -1.7 | -1.2 | -2.4 |
|  | NM_008394.2 | Isgf3g |  | Interferon dependent positive acting transcription factor 3 gamma | -1.53 | -1.9 | -1.1 | -1.7 |
|  | NM_009515.1 | Was |  | Wiskott-Aldrich syndrome homolog (human) | -1.5 | -1.8 | -1.5 | -1.2 |
|  | NM_007778.1 | Csf1 |  | Colony stimulating factor 1 (macrophage) | -1.49 | -1.4 | -1.4 | -1.7 |
|  | NM_013810.1 | Dbnl |  | Drebrin-like | -1.4 | -1.5 | -1 | -1.7 |
|  | NM_009778.1 | C3 | ***** | **Complement component 3** | -1.4 | -1.4 | -0.8 | -2 |
|  | NM_018851.2 | Samhd1 |  | SAM domain and HD domain, 1 | -1.26 | -1.6 | -0.7 | -1.5 |
|  | NM_011336.1 | Ccl27 | ***** | **RIKEN cDNA 1700008B15 gene** | -1.24 | -1.2 | -0.6 | -1.9 |
|  | NM_008689.1 | Nfkb1 |  | Nuclear factor of kappa light chain gene enhancer in B-cells 1, p105 | -1.14 | -1 | -1.4 | -1 |
|  | NM_020022.2 | Rfc2 |  | Replication factor C (activator 1) 2 | -1.14 | -1 | -0.8 | -1.6 |
| **Ribosome** | | | | | | | | |
|  | NM_011029.2 | Lamr1 |  | Laminin receptor 1 (ribosomal protein SA) | -2.25 | -2.1 | -2.2 | -2.5 |
|  | NM_018860.2 | Rpl41 |  | RIKEN cDNA 2210411K19 gene | -1.96 | -1.7 | -1.3 | -2.9 |
|  | NM_011289.1 | Rpl27 | ***** | **Ribosomal protein L27** | -1.93 | -2.1 | -0.9 | -2.8 |
|  | NM_016844.1 | Rps28 | ***** | **Ribosomal protein S28** | -1.85 | -1.9 | -0.7 | -2.9 |
|  | NM_009076.1 | Rpl12 |  | Ribosomal protein L12 | -1.57 | -1.6 | -0.9 | -2.2 |
|  | NM_026020.2 | Rplp2 |  | Ribosomal protein, large P2 | -1.32 | -1.1 | -1.7 | -1.2 |
|  | NM_052835.1 | Rpl10 |  | Ribosomal protein 10 | -1.07 | -1 | -0.7 | -1.5 |
|  | BC091759.1 | Rpl17 | ***** | **Ribosomal protein L17** | -1.01 | -0.8 | -0.6 | -1.6 |
| **Transport** | | | | | | | | |
|  | **Glucose transport** | | | | | | | |
|  | NM_009204.1 | Slc2a4 |  | Solute carrier family 2 (facilitated glucose transporter), member 4 | -1.77 | -2.1 | -2 | -1.2 |
|  | NM_011400.1 | Slc2a1 |  | Solute carrier family 2 (facilitated glucose transporter), member 1 | -1.52 | -1.4 | -1.8 | -1.4 |
|  | NM_019488.3 | Slc2a8 |  | Solute carrier family 2, (facilitated glucose transporter), member 8 | -1.28 | -1.7 | -1 | -1.2 |

Footnotes: M values correspond to the log_2_ gene expression ratio (HFD, LC, MC or HC sample over NCC sample). M values for the HFD group (M HFD) resulted from the “Grouped analysis”. Individual M values for LC (M LC), MC (M MC) or HC (M HC) groups are also indicated. Data are expressed as the mean of M values calculated for each gene in each group (number of mice: n=12 for HFD group and n=4 for LC, MC or HC mice). * and bold: indicate genes that were significantly regulated by rimonabant treatment in the H group.
