# Supplementary material for: Different Transcriptional Control of Metabolism and Extracellular Matrix in Visceral and Subcutaneous Fat of Obese and Rimonabant Treated Mice
Source: PLoS One. 2008 Oct 13;3(10):e3385. doi: 10.1371/journal.pone.0003385 (PMC2586343; doi:10.1371/journal.pone.0003385)
Supplement: Table S3 — (0.32 MB DOC) [file pone.0003385.s003.doc]

**Table S3: List of leading edge genes associated with the genesets of Table 2.**

| **Tissue** | **Comparison** | **Gene sets** | **Gene symbol** | **Annotation** | **moderated t** |
| --- | --- | --- | --- | --- | --- |
| **VAT** | **NCC vs HC** | ***ST_p38_MAPK_Pathway : p38 is a MAP kinase regulated by cytokines and cellular stress.*** | | | |
|  |  |  | Dusp10 | Dual specificity phosphatase 10 | 2.26 |
|  |  |  | Map3k7ip2 | Mitogen-activated protein kinase kinase kinase 7 interacting protein 2 | 2.08 |
|  |  |  | Map3k7 | Mitogen activated protein kinase kinase kinase 7 | 1.98 |
|  |  |  | Cdc42 | Cell division cycle 42 homolog (S. cerevisiae) | 1.60 |
|  |  |  | Il1r1 | Interleukin 1 receptor, type I | 1.42 |
|  |  |  | Eif4e | Eukaryotic translation initiation factor 4E | 1.27 |
|  |  |  | Traf6 | Tnf receptor-associated factor 6 | 1.25 |
|  |  |  | Creb3 | CAMP responsive element binding protein 3 | 1.21 |
|  |  |  | Atf1 | Activating transcription factor 1 | 1.18 |
|  |  |  | Map2k4 | Mitogen activated protein kinase kinase 4 | 1.17 |
|  |  |  | Mapk1 | Mitogen activated protein kinase 1 | 1.10 |
|  |  |  | Hspb1 | Heat shock protein 1 | 1.02 |
|  |  |  |  |  |  |
|  |  | ***ST_JNK_MAPK_Pathway : JNKs are MAP kinases regulated by several levels of kinases (MAPKK, MAPKKK) and phosphorylate transcription factors and regulatory proteins.*** | | | |
|  |  |  | Nfatc3 | Nuclear factor of activated T-cells, cytoplasmic, calcineurin-dependent 3 | 3.94 |
|  |  |  | Mapk9 | Mitogen activated protein kinase 9 | 2.53 |
|  |  |  | Mapk8 | Mitogen activated protein kinase 8 | 2.27 |
|  |  |  | Dusp10 | Dual specificity phosphatase 10 | 2.26 |
|  |  |  | Map3k7ip2 | Mitogen-activated protein kinase kinase kinase 7 interacting protein 2 | 2.08 |
|  |  |  | Gab1 | RIKEN cDNA 1700019H22 gene | 2.06 |
|  |  |  | Map3k7 | Mitogen activated protein kinase kinase kinase 7 | 1.98 |
|  |  |  | Cdc42 | Cell division cycle 42 homolog (S. cerevisiae) | 1.60 |
|  |  |  | Il1r1 | Interleukin 1 receptor, type I | 1.42 |
|  |  |  | Dld | Dihydrolipoamide dehydrogenase | 1.30 |
|  |  |  | Traf6 | Tnf receptor-associated factor 6 | 1.25 |
|  |  |  | Map2k4 | Mitogen activated protein kinase kinase 4 | 1.17 |
|  |  |  | Atf2 | Activating transcription factor 2 | 0.96 |
|  |  |  |  |  |  |
|  |  | ***ST_Differentiation_Pathway_in_PC12_Cells : Rat-derived PC12 cells respond to nerve growth factor (NGF) and PACAP to differentiate into neuronal cells.*** | | | |
|  |  |  | Frs2 | Fibroblast growth factor receptor substrate 2 | 4.48 |
|  |  |  | Rps6ka3 | Hypothetical protein C130006E23 | 3.69 |
|  |  |  | Pik3ca | Phosphatidylinositol 3-kinase, catalytic, alpha polypeptide | 3.06 |
|  |  |  | Tuba1 | Tubulin, alpha 1 | 3.00 |
|  |  |  | Mapk9 | Mitogen activated protein kinase 9 | 2.53 |
|  |  |  | Mapk8 | Mitogen activated protein kinase 8 | 2.27 |
|  |  |  | Ptpn11 | Protein tyrosine phosphatase, non-receptor type 11 | 2.27 |
|  |  |  | Asah1 | N-acylsphingosine amidohydrolase 1 | 2.11 |
|  |  |  | Creb3 | CAMP responsive element binding protein 3 | 1.21 |
|  |  |  | Atf1 | Activating transcription factor 1 | 1.18 |
|  |  |  | Map2k4 | Mitogen activated protein kinase kinase 4 | 1.17 |
|  |  |  | Mapk1 | Mitogen activated protein kinase 1 | 1.10 |
|  |  |  | Dag1 | Dystroglycan 1 | 0.88 |
|  |  |  | Pik3r1 | Phosphatidylinositol 3-kinase, regulatory subunit, polypeptide 1 (p85 alpha) | 0.77 |
|  |  |  | Mapk10 | Mitogen activated protein kinase 10 | 0.74 |
|  |  |  |  |  |  |
|  |  | ***CR_CAM : Cancer related genes involved in cell adhesion and metalloproteinases.*** | | | |
|  |  |  | Itgb1 | Integrin beta 1 (fibronectin receptor beta) | 4.73 |
|  |  |  | Itgav | Integrin alpha V | 4.51 |
|  |  |  | Arhgap6 | Rho GTPase activating protein 6 | 3.30 |
|  |  |  | Rock1 | Rho-associated coiled-coil forming kinase 1 | 2.81 |
|  |  |  | Wasl | Wiskott-Aldrich syndrome-like (human) | 2.78 |
|  |  |  | Timp2 | Tissue inhibitor of metalloproteinase 2 | 2.64 |
|  |  |  | Timp3 | Tissue inhibitor of metalloproteinase 3 | 2.60 |
|  |  |  | Fath | Fat tumor suppressor homolog (Drosophila) | 2.55 |
|  |  |  | Cd47 | CD47 antigen (Rh-related antigen, integrin-associated signal transducer) | 2.41 |
|  |  |  | Mmp11 | Matrix metalloproteinase 11 | 2.28 |
|  |  |  | Fn1 | Fibronectin 1 | 2.22 |
|  |  |  | Rac1 | RAS-related C3 botulinum substrate 1 | 2.16 |
|  |  |  | Cdh5 | Cadherin 5 | 2.06 |
|  |  |  | Cdh11 | Cadherin 11 | 2.02 |
|  |  |  | Neo1 | Neogenin | 1.98 |
|  |  |  | Ptk2 | PTK2 protein tyrosine kinase 2 | 1.37 |
|  |  |  | Jup | Junction plakoglobin | 1.31 |
|  |  |  | Cd44 | CD44 antigen | 1.30 |
|  |  |  | Itga9 | Integrin alpha 9 | 1.12 |
|  |  |  | Itgb5 | Integrin beta 5 | 0.99 |
|  |  |  | Pecam1 | Platelet/endothelial cell adhesion molecule 1 | 0.90 |
|  |  |  | Itga7 | Integrin alpha 7 | 0.80 |
|  |  |  | Cd97 | CD97 antigen | 0.79 |
|  |  |  | Mcam | Melanoma cell adhesion molecule | 0.72 |
|  |  |  | Alcam | Activated leukocyte cell adhesion molecule | 0.66 |
|  |  |  |  |  |  |
|  |  | ***EMT_UP : Genes upregulated for epithelial plasticity in tumor progression.*** | | | |
|  |  |  | Mmp12 | Matrix metalloproteinase 12 | 5.21 |
|  |  |  | Vldlr | Very low density lipoprotein receptor | 4.71 |
|  |  |  | Sparc | Secreted acidic cysteine rich glycoprotein | 4.53 |
|  |  |  | Vim | Vimentin | 4.23 |
|  |  |  | Dab2 | Disabled homolog 2 (Drosophila) | 3.76 |
|  |  |  | Cmkor1 | Chemokine orphan receptor 1 | 3.66 |
|  |  |  | Pdgfra | Platelet derived growth factor receptor, alpha polypeptide | 3.63 |
|  |  |  | Snai1 | Snail homolog 1 (Drosophila) | 3.19 |
|  |  |  | Sdc2 | RIKEN cDNA 4833414L08 gene | 2.93 |
|  |  |  | Hif1a | Hypoxia inducible factor 1, alpha subunit | 2.85 |
|  |  |  | Dcn | Decorin | 2.63 |
|  |  |  | Pla2g7 | Phospholipase A2, group VII (platelet-activating factor acetylhydrolase, plasma) | 2.44 |
|  |  |  | Col6a1 | Procollagen, type VI, alpha 1 | 2.20 |
|  |  |  |  |  |  |
|  |  | ***SFT : Solitary Fibrous Tumor (gene set with a strong mesenchymal stem cell component)*** | | | |
|  |  |  | Itpr2 | Inositol 1,4,5-triphosphate receptor 2 | 3.99 |
|  |  |  | Cd36 | CD36 antigen | 3.97 |
|  |  |  | Gsn | Gelsolin | 3.77 |
|  |  |  | Nr3c1 | Nuclear receptor subfamily 3, group C, member 1 | 3.56 |
|  |  |  | Ncoa2 | Nuclear receptor coactivator 2 | 3.40 |
|  |  |  | Arhgap6 | Rho GTPase activating protein 6 | 3.30 |
|  |  |  | Atrnl1 | RIKEN cDNA E430016L07 gene | 3.13 |
|  |  |  | D430039N05Rik | RIKEN cDNA D430039N05 gene | 2.93 |
|  |  |  | Slc40a1 | Solute carrier family 40 (iron-regulated transporter), member 1 | 2.82 |
|  |  |  | Sorbs1 | Sorbin and SH3 domain containing 1 | 2.77 |
|  |  |  | Aldh1a1 | RIKEN cDNA E030003E18 gene | 2.76 |
|  |  |  | Nab1 | Ngfi-A binding protein 1 | 2.72 |
|  |  |  | Bbs2 | Bardet-Biedl syndrome 2 homolog (human) | 2.40 |
|  |  |  | Tpr | Translocated promoter region | 2.31 |
|  |  |  | Col4a5 | Procollagen, type IV, alpha 5 | 2.16 |
|  |  |  | Txnip | Thioredoxin interacting protein | 2.11 |
|  |  |  | Dpp8 | Dipeptidylpeptidase 8 | 1.64 |
|  |  |  | Bcl2 | B-cell leukemia/lymphoma 2 | 1.38 |
|  |  |  | Ppap2b | Phosphatidic acid phosphatase type 2B | 1.38 |
|  |  |  | Abhd2 | Abhydrolase domain containing 2 | 1.30 |
|  |  |  | Tle3 | Transducin-like enhancer of split 3, homolog of Drosophila E(spl) | 1.24 |
|  |  |  | Ssx2ip | Synovial sarcoma, X breakpoint 2 interacting protein | 1.19 |
|  |  |  | F3 | Coagulation factor III | 1.12 |
|  |  |  | Prkcd | Protein kinase C, delta | 1.09 |
|  |  |  | Usp32 | Ubiquitin specific protease 32 | 0.98 |
|  |  |  | Fnta | Farnesyltransferase, CAAX box, alpha | 0.93 |
|  |  |  | Itm2a | Integral membrane protein 2A | 0.92 |
|  |  |  | Fmn2 | Formin 2 | 0.90 |
|  |  |  | Odz4 | Odd Oz/ten-m homolog 4 (Drosophila) | 0.87 |
|  |  |  | Stat6 | Signal transducer and activator of transcription 6 | 0.87 |
|  |  |  | G430041M01Rik | RIKEN cDNA G430041M01 gene | 0.80 |
|  |  |  | Mapk10 | Mitogen activated protein kinase 10 | 0.74 |
|  |  |  | Btg2 | B-cell translocation gene 2, anti-proliferative | 0.69 |
|  |  |  |  |  |  |
|  | **NCT vs HT** | ***mRNA splicing*** | | | |
|  |  |  | Ivns1abp | Influenza virus NS1A binding protein | 1.95 |
|  |  |  | U2af1-rs2 | U2 small nuclear ribonucleoprotein auxiliary factor (U2AF) 1, related sequence 2 | 1.82 |
|  |  |  | Srp19 | Signal recognition particle 19 | 1.65 |
|  |  |  | Snrpa1 | Small nuclear ribonucleoprotein polypeptide A' | 1.63 |
|  |  |  | Sf3a3 | Splicing factor 3a, subunit 3 | 1.47 |
|  |  |  | Sf3b3 | Splicing factor 3b, subunit 3 | 1.22 |
|  |  |  | Snrpb2 | U2 small nuclear ribonucleoprotein B | 1.08 |
|  |  |  | Sfrs7 | Splicing factor, arginine/serine-rich 7 | 1.06 |
|  |  |  | Hnrpc | Heterogeneous nuclear ribonucleoprotein C | 1.03 |
|  |  |  | Sfrs10 | RIKEN cDNA 5730405G21 gene | 0.98 |
|  |  |  | Snrpg | Small nuclear ribonucleoprotein polypeptide G | 0.69 |
|  |  |  | Snrp1c | U1 small nuclear ribonucleoprotein 1C | 0.65 |
|  |  |  | Sfrs11 | Splicing factor, arginine/serine-rich 11 | 0.54 |
|  |  |  | Sfrs2 | Splicing factor, arginine/serine-rich 2 (SC-35) | 0.50 |
|  |  |  | Smndc1 | Survival motor neuron domain containing 1 | 0.39 |
|  |  |  | Prpf3 | PRP3 pre-mRNA processing factor 3 homolog (yeast) | 0.20 |
|  |  |  | Nono | Non-POU-domain-containing, octamer binding protein | 0.13 |
|  |  |  | Sfpq | RIKEN cDNA 1110004P21 gene | 0.06 |
|  |  |  | Prpf4 | PRP4 pre-mRNA processing factor 4 homolog (yeast) | -0.08 |
|  |  |  | Ppig | Peptidyl-prolyl isomerase G (cyclophilin G) | -0.08 |
|  |  |  | Prpf8 | Pre-mRNA processing factor 8 | -0.09 |
|  |  |  | Fmnl3 | Formin-like 3 | -0.10 |
|  |  |  | Sfrs2ip | Splicing factor, arginine/serine-rich 2, interacting protein | -0.11 |
|  |  |  |  |  |  |
| **SCAT** | **NCC vs HC** | ***MAP00561_Glycerolipid_metabolism : Glycerolipid Metabolism (KEGG).*** | | | |
|  |  |  | Aldh1a1 | RIKEN cDNA E030003E18 gene | 5.93 |
|  |  |  | Lpl | Lipoprotein lipase | 3.84 |
|  |  |  | Pcyt1a | Phosphate cytidylyltransferase 1, choline, alpha isoform | 3.62 |
|  |  |  | Dgke | Diacylglycerol kinase, epsilon | 3.31 |
|  |  |  | Cpt1a | Carnitine palmitoyltransferase 1a, liver | 3.12 |
|  |  |  | Gyk | Glycerol kinase | 2.23 |
|  |  |  | Pafah1b1 | Platelet-activating factor acetylhydrolase, isoform 1b, beta1 subunit | 2.09 |
|  |  |  | Aldh3a2 | Aldehyde dehydrogenase family 3, subfamily A2 | 1.99 |
|  |  |  | Agps | Alkylglycerone phosphate synthase | 1.50 |
|  |  |  | Adh5 | Alcohol dehydrogenase 5 (class III), chi polypeptide | 1.30 |
|  |  |  |  |  |  |
|  |  | ***RAR_UP : Genes upregulated by retinoic acid receptor.*** | | | |
|  |  |  | Snx10 | Sorting nexin 10 | 4.02 |
|  |  |  | Dcn | Decorin | 2.71 |
|  |  |  | Lamp2 | Lysosomal membrane glycoprotein 2 | 2.37 |
|  |  |  | Lbp | Lipopolysaccharide binding protein | 2.14 |
|  |  |  | Ctsb | Cathepsin B | 2.11 |
|  |  |  | Net1 | Neuroepithelial cell transforming gene 1 | 1.97 |
|  |  |  | Abca1 | ATP-binding cassette, sub-family A (ABC1), member 1 | 1.87 |
|  |  |  | Ssr1 | Signal sequence receptor, alpha | 1.67 |
|  |  |  | Anxa6 | Annexin A6 | 1.33 |
|  |  |  | Marcks | Myristoylated alanine rich protein kinase C substrate | 1.20 |
|  |  |  | Col4a5 | Procollagen, type IV, alpha 5 | 1.17 |
|  |  |  | Slc12a2 | Solute carrier family 12, member 2 | 1.04 |
|  |  |  |  |  |  |
|  |  | ***ST_JNK_MAPK_Pathway : JNKs are MAP kinases regulated by several levels of kinases (MAPKK, MAPKKK) and phosphorylate transcription factors and regulatory proteins.*** | | | |
|  |  |  | Dld | Dihydrolipoamide dehydrogenase | 3.45 |
|  |  |  | Map3k7ip2 | Mitogen-activated protein kinase kinase kinase 7 interacting protein 2 | 1.84 |
|  |  |  | Cdc42 | Cell division cycle 42 homolog (S. cerevisiae) | 1.82 |
|  |  |  | Map3k7 | Mitogen activated protein kinase kinase kinase 7 | 1.21 |
|  |  |  | Gadd45a | Growth arrest and DNA-damage-inducible 45 alpha | 1.03 |
|  |  |  | Dusp10 | Dual specificity phosphatase 10 | 0.93 |
|  |  |  | Mapk8 | Mitogen activated protein kinase 8 | 0.84 |
|  |  |  | Nr2c2 | Nuclear receptor subfamily 2, group C, member 2 | 0.74 |
|  |  |  | Gab1 | RIKEN cDNA 1700019H22 gene | 0.45 |
|  |  |  | Atf2 | Activating transcription factor 2 | 0.39 |
|  |  |  | Mapk10 | Mitogen activated protein kinase 10 | -0.06 |
|  |  |  | Mapk9 | Mitogen activated protein kinase 9 | -0.13 |
|  |  |  | Jun | Jun oncogene | -0.21 |
|  |  |  | Map3k3 | Mitogen activated protein kinase kinase kinase 3 | -0.36 |
|  |  |  | Shc1 | Src homology 2 domain-containing transforming protein C1 | -0.44 |
|  |  |  |  |  |  |
|  |  | ***ST_Differentiation_Pathway_in_PC12_Cells : Rat-derived PC12 cells respond to nerve growth factor (NGF) and PACAP to differentiate into neuronal cells.*** | | | |
|  |  |  | Rps6ka3 | Hypothetical protein C130006E23 | 3.40 |
|  |  |  | Ptpn11 | Protein tyrosine phosphatase, non-receptor type 11 | 3.23 |
|  |  |  | Pik3r1 | Phosphatidylinositol 3-kinase, regulatory subunit, polypeptide 1 (p85 alpha) | 2.31 |
|  |  |  | Tuba1 | Tubulin, alpha 1 | 1.92 |
|  |  |  | Pik3ca | Phosphatidylinositol 3-kinase, catalytic, alpha polypeptide | 1.88 |
|  |  |  | Asah1 | N-acylsphingosine amidohydrolase 1 | 1.67 |
|  |  |  | Frs2 | Fibroblast growth factor receptor substrate 2 | 1.33 |
|  |  |  | Atf1 | Activating transcription factor 1 | 1.03 |
|  |  |  | Dag1 | Dystroglycan 1 | 1.01 |
|  |  |  | Mapk8 | Mitogen activated protein kinase 8 | 0.84 |
|  |  |  | Mapk1 | Mitogen activated protein kinase 1 | 0.83 |
|  |  |  | Creb3 | CAMP responsive element binding protein 3 | 0.73 |
|  |  |  | Crebbp | CREB binding protein | 0.53 |
|  |  |  | Mapk10 | Mitogen activated protein kinase 10 | -0.06 |
|  |  |  | Mapk9 | Mitogen activated protein kinase 9 | -0.13 |
|  |  |  | Jun | Jun oncogene | -0.21 |
|  |  |  | Mapk3 | Mitogen activated protein kinase 3 | -0.30 |
|  |  |  | Shc1 | Src homology 2 domain-containing transforming protein C1 | -0.44 |
|  |  |  | Egr1 | Early growth response 1 | -0.58 |
|  |  |  |  |  |  |
|  | **NCT vs HT** | ***GLUCOSE_DOWN : Genes down-regulated by glucose starvation.*** | | | |
|  |  |  | Mtap4 | Microtubule-associated protein 4 | 2.27 |
|  |  |  | Slc12a4 | Solute carrier family 12, member 4 | 2.02 |
|  |  |  | Ilk | Integrin linked kinase | 1.83 |
|  |  |  | Uchl1 | Ubiquitin carboxy-terminal hydrolase L1 | 1.81 |
|  |  |  | Txnip | Thioredoxin interacting protein | 1.77 |
|  |  |  | Rap1a | RAS-related protein-1a | 1.64 |
|  |  |  | Rnf6 | Ring finger protein (C3H2C3 type) 6 | 1.57 |
|  |  |  | AI462438 | Expressed sequence AI462438 | 1.53 |
|  |  |  | Dyrk1a | Dual-specificity tyrosine-(Y)-phosphorylation regulated kinase 1a | 1.44 |
|  |  |  | Rbbp4 | Retinoblastoma binding protein 4 | 1.40 |
|  |  |  | Atp2a2 | ATPase, Ca++ transporting, cardiac muscle, slow twitch 2 | 1.38 |
|  |  |  | Nap1l1 | Nucleosome assembly protein 1-like 1 | 1.36 |
|  |  |  | Nfyb | Nuclear transcription factor-Y beta | 1.30 |
|  |  |  | Cd47 | CD47 antigen (Rh-related antigen, integrin-associated signal transducer) | 1.12 |
|  |  |  | Rab5a | RAB5A, member RAS oncogene family | 1.08 |
|  |  |  | Fosl2 | Fos-like antigen 2 | 1.07 |
|  |  |  | Eif3s3 | Eukaryotic translation initiation factor 3, subunit 3 (gamma) | 1.05 |
|  |  |  | Maged1 | Melanoma antigen, family D, 1 | 1.04 |
|  |  |  | Purb | Purine rich element binding protein B | 1.04 |
|  |  |  | Tegt | Testis enhanced gene transcript | 1.02 |
|  |  |  | Ehd1 | EH-domain containing 1 | 0.94 |
|  |  |  | Ssbp1 | Single-stranded DNA binding protein 1 | 0.93 |
|  |  |  | Usp33 | Ubiquitin specific protease 33 | 0.84 |
|  |  |  | Polg | Polymerase (DNA directed), gamma | 0.84 |
|  |  |  | Sec24c | SEC24 related gene family, member C (S. cerevisiae) | 0.81 |
|  |  |  | Prkcbp1 | Protein kinase C binding protein 1 | 0.78 |
|  |  |  | Wbscr1 | Williams-Beuren syndrome chromosome region 1 homolog (human) | 0.77 |
|  |  |  | Asf1a | ASF1 anti-silencing function 1 homolog A (S. cerevisiae) | 0.74 |
|  |  |  | Mad2l1 | MAD2 (mitotic arrest deficient, homolog)-like 1 (yeast) | 0.69 |
|  |  |  | Phb | Prohibitin | 0.61 |
|  |  |  | E2f1 | E2F transcription factor 1 | 0.60 |
|  |  |  | Picalm | Phosphatidylinositol binding clathrin assembly protein | 0.59 |
|  |  |  | Msh2 | MutS homolog 2 (E. coli) | 0.59 |
|  |  |  | Dpysl2 | Dihydropyrimidinase-like 2 | 0.56 |
|  |  |  | Zfr | Zinc finger RNA binding protein | 0.53 |
|  |  |  | AW555464 | Expressed sequence AW555464 | 0.49 |
|  |  |  | Pdcd4 | Programmed cell death 4 | 0.46 |
|  |  |  | Arf4 | ADP-ribosylation factor 4 | 0.44 |
|  |  |  | Fubp1 | Far upstream element (FUSE) binding protein 1 | 0.42 |
|  |  |  | Cct4 | Chaperonin subunit 4 (delta) | 0.42 |
|  |  |  | Psma5 | Proteasome (prosome, macropain) subunit, alpha type 5 | 0.36 |
|  |  |  | Gorasp2 | Golgi reassembly stacking protein 2 | 0.30 |
|  |  |  | Rab9 | RAB9, member RAS oncogene family | 0.26 |
|  |  |  | Ell | Elongation factor RNA polymerase II | 0.24 |
|  |  |  | Dnaja1 | DnaJ (Hsp40) homolog, subfamily A, member 1 | 0.24 |
|  |  |  | Dek | DEK oncogene (DNA binding) | 0.21 |
|  |  |  | Arpc5 | Actin related protein 2/3 complex, subunit 5 | 0.17 |
|  |  |  | Sfrs1 | Splicing factor, arginine/serine-rich 1 (ASF/SF2) | 0.17 |
|  |  |  | Slc25a5 | Solute carrier family 25 (mitochondrial carrier, adenine nucleotide translocator), member 5 | 0.16 |
|  |  |  | Cdc2a | Cell division cycle 2 homolog A (S. pombe) | 0.16 |
|  |  |  | Capza1 | Capping protein (actin filament) muscle Z-line, alpha 1 | 0.15 |
|  |  |  |  |  |  |
|  |  | ***VOXPHOS : Oxidative Phosphorylation.*** | | | |
|  |  |  | Ndufc1 | NADH dehydrogenase (ubiquinone) 1, subcomplex unknown, 1 | -3.53 |
|  |  |  | Cox4i1 | Cytochrome c oxidase subunit IV isoform 1 | -3.02 |
|  |  |  | Atp5j2 | ATP synthase, H+ transporting, mitochondrial F0 complex, subunit f, isoform 2 | -2.99 |
|  |  |  | Atp5g1 | ATP synthase, H+ transporting, mitochondrial F0 complex, subunit c (subunit 9), isoform 1 | -2.90 |
|  |  |  | Uqcr | Ubiquinol-cytochrome c reductase (6.4kD) subunit | -2.89 |
|  |  |  | Cox6a1 | Cytochrome c oxidase, subunit VI a, polypeptide 1 | -2.62 |
|  |  |  | Atp5o | ATP synthase, H+ transporting, mitochondrial F1 complex, O subunit | -2.55 |
|  |  |  | Pet112l | PET112-like (yeast) | -2.49 |
|  |  |  | Ndufb7 | NADH dehydrogenase (ubiquinone) 1 beta subcomplex, 7 | -2.48 |
|  |  |  | Atp5l | ATP synthase, H+ transporting, mitochondrial F0 complex, subunit g | -2.45 |
|  |  |  | Ndufs6 | NADH dehydrogenase (ubiquinone) Fe-S protein 6 | -2.44 |
|  |  |  | Atp5k | Hypothetical protein D830035I06 | -2.34 |
|  |  |  | Ndufb5 | NADH dehydrogenase (ubiquinone) 1 beta subcomplex, 5 | -2.33 |
|  |  |  | Atp5g3 | ATP synthase, H+ transporting, mitochondrial F0 complex, subunit c (subunit 9), isoform 3 | -2.25 |
|  |  |  | Cox6c | V-src suppressed transcript 3 | -2.14 |
|  |  |  | Sdhc | Succinate dehydrogenase complex, subunit C, integral membrane protein | -1.96 |
|  |  |  | Cox7c | Cytochrome c oxidase, subunit VIIc | -1.90 |
|  |  |  | Ndufs5 | NADH dehydrogenase (ubiquinone) Fe-S protein 5 | -1.87 |
|  |  |  | Atp5c1 | ATP synthase, H+ transporting, mitochondrial F1 complex, gamma polypeptide 1 | -1.73 |
|  |  |  | Ndufa8 | NADH dehydrogenase (ubiquinone) 1 alpha subcomplex, 8 | -1.71 |
|  |  |  | Uqcrh | Ubiquinol-cytochrome c reductase hinge protein | -1.67 |
|  |  |  | Ndufs7 | NADH dehydrogenase (ubiquinone) Fe-S protein 7 | -1.63 |
|  |  |  | Ndufc2 | NADH dehydrogenase (ubiquinone) 1, subcomplex unknown, 2 | -1.59 |
|  |  |  | Atp5d | ATP synthase, H+ transporting, mitochondrial F1 complex, delta subunit | -1.58 |
|  |  |  | Sdhb | Succinate dehydrogenase complex, subunit B, iron sulfur (Ip) | -1.57 |
|  |  |  | Ndufs2 | NADH dehydrogenase (ubiquinone) Fe-S protein 2 | -1.30 |
|  |  |  | Uqcrfs1 | Ubiquinol-cytochrome c reductase, Rieske iron-sulfur polypeptide 1 | -1.29 |
|  |  |  | Cyc1 | Cytochrome c-1 | -1.16 |
|  |  |  | Cox7a2 | Cytochrome c oxidase, subunit VIIa 2 | -1.08 |
|  |  |  | Ndufs8 | NADH dehydrogenase (ubiquinone) Fe-S protein 8 | -0.89 |
|  |  |  | Sdhd | Succinate dehydrogenase complex, subunit D, integral membrane protein | -0.85 |
|  |  |  | Atp5b | ATP synthase, H+ transporting mitochondrial F1 complex, beta subunit | -0.65 |
|  |  |  | Uqcrb | Ubiquinol-cytochrome c reductase binding protein | -0.59 |
|  |  |  | Surf2 | Surfeit gene 2 | -0.58 |
|  |  |  | Atp5f1 | ATP synthase, H+ transporting, mitochondrial F0 complex, subunit b, isoform 1 | -0.53 |
|  |  |  | Bcs1l | BCS1-like (yeast) | -0.46 |
|  |  |  | Uqcrc1 | Ubiquinol-cytochrome c reductase core protein 1 | -0.46 |
|  |  |  | Cycs | Cytochrome c, somatic | -0.43 |
|  |  |  | Mbd3 | Methyl-CpG binding domain protein 3 | -0.43 |
|  |  |  |  |  |  |
|  |  | ***Electron_Transport_Chain : electron transport chain.*** | | | |
|  |  |  | Ndufc1 | NADH dehydrogenase (ubiquinone) 1, subcomplex unknown, 1 | -3.53 |
|  |  |  | Ndufb9 | NADH dehydrogenase (ubiquinone) 1 beta subcomplex, 9 | -3.07 |
|  |  |  | Ndufb10 | NADH dehydrogenase (ubiquinone) 1 beta subcomplex, 10 | -3.06 |
|  |  |  | Cox4i1 | Cytochrome c oxidase subunit IV isoform 1 | -3.02 |
|  |  |  | Atp5j2 | ATP synthase, H+ transporting, mitochondrial F0 complex, subunit f, isoform 2 | -2.99 |
|  |  |  | Atp5g1 | ATP synthase, H+ transporting, mitochondrial F0 complex, subunit c (subunit 9), isoform 1 | -2.90 |
|  |  |  | Uqcr | Ubiquinol-cytochrome c reductase (6.4kD) subunit | -2.89 |
|  |  |  | Cox6a1 | Cytochrome c oxidase, subunit VI a, polypeptide 1 | -2.62 |
|  |  |  | Atp5o | ATP synthase, H+ transporting, mitochondrial F1 complex, O subunit | -2.55 |
|  |  |  | Ndufb7 | NADH dehydrogenase (ubiquinone) 1 beta subcomplex, 7 | -2.48 |
|  |  |  | Atp5l | ATP synthase, H+ transporting, mitochondrial F0 complex, subunit g | -2.45 |
|  |  |  | Ndufs6 | NADH dehydrogenase (ubiquinone) Fe-S protein 6 | -2.44 |
|  |  |  | Atp5k | Hypothetical protein D830035I06 | -2.34 |
|  |  |  | Ndufb5 | NADH dehydrogenase (ubiquinone) 1 beta subcomplex, 5 | -2.33 |
|  |  |  | Atp5g3 | ATP synthase, H+ transporting, mitochondrial F0 complex, subunit c (subunit 9), isoform 3 | -2.25 |
|  |  |  | Cox6c | V-src suppressed transcript 3 | -2.14 |
|  |  |  | Popdc2 | Popeye domain containing 2 | -2.10 |
|  |  |  | Sdhc | Succinate dehydrogenase complex, subunit C, integral membrane protein | -1.96 |
|  |  |  | Cox7c | Cytochrome c oxidase, subunit VIIc | -1.90 |
|  |  |  | Ndufs5 | NADH dehydrogenase (ubiquinone) Fe-S protein 5 | -1.87 |
|  |  |  | Atp5c1 | ATP synthase, H+ transporting, mitochondrial F1 complex, gamma polypeptide 1 | -1.73 |
|  |  |  | Ndufa8 | NADH dehydrogenase (ubiquinone) 1 alpha subcomplex, 8 | -1.71 |
|  |  |  | Uqcrh | Ubiquinol-cytochrome c reductase hinge protein | -1.67 |
|  |  |  | Ndufs7 | NADH dehydrogenase (ubiquinone) Fe-S protein 7 | -1.63 |
|  |  |  | Ndufc2 | NADH dehydrogenase (ubiquinone) 1, subcomplex unknown, 2 | -1.59 |
|  |  |  | Atp5d | ATP synthase, H+ transporting, mitochondrial F1 complex, delta subunit | -1.58 |
|  |  |  | Sdhb | Succinate dehydrogenase complex, subunit B, iron sulfur (Ip) | -1.57 |
|  |  |  | Ndufs2 | NADH dehydrogenase (ubiquinone) Fe-S protein 2 | -1.30 |
|  |  |  | Uqcrfs1 | Ubiquinol-cytochrome c reductase, Rieske iron-sulfur polypeptide 1 | -1.29 |
|  |  |  | Cox7a2 | Cytochrome c oxidase, subunit VIIa 2 | -1.08 |
|  |  |  | Ndufs8 | NADH dehydrogenase (ubiquinone) Fe-S protein 8 | -0.89 |
|  |  |  | Sdhd | Succinate dehydrogenase complex, subunit D, integral membrane protein | -0.85 |
|  |  |  | Atp5b | ATP synthase, H+ transporting mitochondrial F1 complex, beta subunit | -0.65 |
|  |  |  | Uqcrb | Ubiquinol-cytochrome c reductase binding protein | -0.59 |
|  |  |  | Atp5f1 | ATP synthase, H+ transporting, mitochondrial F0 complex, subunit b, isoform 1 | -0.53 |
|  |  |  | Uqcrc1 | Ubiquinol-cytochrome c reductase core protein 1 | -0.46 |
|  |  |  | Cycs | Cytochrome c, somatic | -0.43 |
|  |  |  | Ndufv1 | NADH dehydrogenase (ubiquinone) flavoprotein 1 | -0.39 |
|  |  |  |  |  |  |
|  |  | ***ST_B_Cell_Antigen_Receptor : B cell receptors bind antigens and promote B cell activation.*** | | | |
|  |  |  | Pik3cd | Phosphatidylinositol 3-kinase catalytic delta polypeptide | -2.60 |
|  |  |  | Shc1 | Src homology 2 domain-containing transforming protein C1 | -2.08 |
|  |  |  | Akt2 | Thymoma viral proto-oncogene 2 | -1.78 |
|  |  |  | Pik3r1 | Phosphatidylinositol 3-kinase, regulatory subunit, polypeptide 1 (p85 alpha) | -1.47 |
|  |  |  | Blnk | B-cell linker | -1.32 |
|  |  |  | Csk | C-src tyrosine kinase | -1.04 |
|  |  |  | Lyn | Yamaguchi sarcoma viral (v-yes-1) oncogene homolog | -0.90 |
|  |  |  | Pik3ca | Phosphatidylinositol 3-kinase, catalytic, alpha polypeptide | -0.79 |
|  |  |  | Nfkb1 | Ubiquitin-conjugating enzyme E2D 3 (UBC4/5 homolog, yeast) | -0.76 |
|  |  |  | Raf1 | V-raf-1 leukemia viral oncogene 1 | -0.74 |

Footnotes: moderated-t: the statistic was calculated using the limma package from Bioconductor to rank genes in the GSEA procedure.
