# Supplementary material for: Different Transcriptional Control of Metabolism and Extracellular Matrix in Visceral and Subcutaneous Fat of Obese and Rimonabant Treated Mice
Source: PLoS One. 2008 Oct 13;3(10):e3385. doi: 10.1371/journal.pone.0003385 (PMC2586343; doi:10.1371/journal.pone.0003385)
Supplement: Table S4 — (0.21 MB DOC) [file pone.0003385.s004.doc]

**Table S4. Gene expression level in the VAT of H group mice treated with vehicle (Veh) 0r Rimonabant (Rimo). The effect of Rimonabant is indicated as “Diff M) and whether the genes expression level was correlated with body weight is indicated in the Ob. Link column, with 1 indicating presence and 0 absence of correlation.**

| **FUNCTIONS** | **GENE SYMB.** | **ANNOTATION** |  | | | |
| --- | --- | --- | --- | --- | --- | --- |
| **Cell adhesion / Cytoskeleton** | | | **Veh.** | **Rimo.** | **Diff. M** | **Ob. Link.** |
| cell adhesion | App | amyloid beta (a4) precursor protein | 1.11 | 0.29 | -0.83 | 1 |
|  | Cd36 | cd36 antigen | 2.7 | 0.91 | -1.79 | 1 |
|  | Cxadr | coxsackievirus and adenovirus receptor | 1.99 | 0.59 | -1.4 | 0 |
|  | Plekhc1 | pleckstrin homology domain containing, family c (with ferm domain) member 1 | 1.17 | 0.3 | -0.87 | 0 |
|  | Stim2 | stromal interaction molecule 2 | 1.31 | 0.37 | -0.93 | 1 |
|  | Vcl | vinculin | 0.94 | 0.17 | -0.77 | 0 |
|  | Nid1 | nidogen 1 | 1.12 | 0.43 | -0.68 | 1 |
|  | Sparc | secreted acidic cysteine rich glycoprotein | 1.56 | 0.24 | -1.32 | 1 |
|  | Adamts1 | a disintegrin-like and metallopeptidse (reprolysin type) with thrombospondin type 1 motif, 1 | 1.39 | 0.35 | -1.04 | 0 |
|  | Itgav | integrin alpha v | 1.13 | 0.44 | -0.69 | 0 |
|  | Itgb1 | integrin beta 1 (fibronectin receptor beta) | 1.67 | 0.21 | -1.47 | 0 |
|  | Atrnl1 | attractin like 1 | 0.94 | -0.01 | -0.95 | 0 |
|  | Ppfibp1 | PTPRF interacting protein, binding protein 1 (liprin beta 1) | 1.07 | 0.10 | -0.97 | 0 |
| cytoskeleton | Actr2 | arp2 actin-related protein 2 homolog (yeast) | 1.29 | 0 | -1.29 | 0 |
|  | Frmd4b | ferm domain containing 4b | 1.09 | 0.22 | -0.87 | 0 |
|  | Spnb2 | spectrin beta 2 | 1.72 | 0.54 | -1.18 | 1 |
|  | Tpm4 | tropomyosin 4 | 1.31 | 0.25 | -1.06 | 1 |
|  | Vim | vimentin | 1.26 | 0.59 | -0.67 | 1 |
|  | Akap2 | a kinase (prka) anchor protein 2 | 1.68 | 0.5 | -1.18 | 1 |
|  | Sestd1 | SEC14 and spectrin domains 1 | 0.89 | -0.04 | -0.93 | 1 |
|  | Anxa2 | Annexin A2 | 1.60 | 0.71 | -0.89 | 1 |
|  | Ivns1abp | influenza virus NS1A binding protein | 2.00 | 0.33 | -1.68 | 0 |
|  | Iqgap1 | IQ motif containing GTPase activating protein 1 | 1.10 | 0.27 | -0.83 | 1 |
|  |  |  |  |  |  |  |
| **Vesicle structure / transport (exocytosis, endocytosis) / function** | | | |  |  |  |
|  | Anxa1 | annexin a1 | 2.61 | 1.18 | -1.43 | 0 |
|  | Cav1 | caveolin, caveolae protein 1 | 2.05 | 0.62 | -1.43 | 1 |
|  | Cav2 | caveolin 2 | 2.19 | 0.62 | -1.57 | 0 |
|  | Lamp2 | lysosomal membrane glycoprotein 2 | 1.6 | 0.43 | -1.17 | 0 |
|  | Wdr7 | WD repeat domain 7 | -0.79 | -0.08 | 0.71 | 0 |
|  | Cltc | clathrin, heavy polypeptide (Hc) | 1.59 | 0.28 | -1.31 | 0 |
|  | Eps15 | epidermal growth factor receptor pathway substrate 15 | 1.36 | 0.25 | -1.11 | 0 |
|  | Dncic2 | dynein, cytoplasmic, intermediate chain 2 | 1.34 | 0.33 | -1.01 | 0 |
|  | Kif5b | kinesin family member 5b | 1 | -0.06 | -1.06 | 0 |
|  |  |  |  |  |  |  |
| **Transport / Chaperone** | |  |  |  |  |  |
|  | Canx | calnexin | 1.06 | 0.01 | -1.05 | 0 |
|  | Sgta | small glutamine-rich tetratricopeptide repeat (tpr)-containing, alpha | -0.86 | -0.17 | 0.69 | 0 |
|  | Hspca | heat shock protein 90kDa alpha (cytosolic), class A member 1 (Hsp90aa1) | 1.51 | -0.38 | -1.89 | 0 |
|  | Dnajc10 | dnaj (hsp40) homolog, subfamily c, member 10 | 0.92 | 0.16 | -0.76 | 0 |
|  | Srp72 | signal recognition particle 72kDa | 1.07 | 0.00 | -1.07 | 0 |
|  | Atp5k | atp synthase, h+ transporting, mitochondrial f1f0 complex, subunit e | -1.25 | 0.15 | 1.39 | 0 |
|  | Clic4 | chloride intracellular channel 4 (mitochondrial) | 1.15 | 0.54 | -0.61 | 1 |
|  | Slc39a8 | solute carrier family 39, member 8 | 1.66 | 0.45 | -1.21 | 0 |
|  | Slc39a10 | solute carrier family 39, member 10 | 1.06 | 0.18 | -0.88 | 0 |
|  | Vldlr | very low density lipoprotein receptor | 1.97 | 0.8 | -1.17 | 0 |
|  | Osbpl8 | oxysterol binding protein-like 8 | 1.38 | 0.2 | -1.18 | 1 |
|  | Copg2 | coatomer protein complex, subunit gamma 2 | 1.11 | 0.25 | -0.86 | 1 |
|  | Sec63 | sec63-like (s. cerevisiae) | 1.34 | 0.16 | -1.19 | 0 |
|  | Tloc1 | sec62: Translocation protein SEC62 | 1.66 | 0.03 | -1.63 | 0 |
|  | Tmed7 | transmembrane emp24 protein transport domain containing 7 | 1.17 | -0.04 | -1.21 | 0 |
|  | Tomm20 | translocase of outer mitochondrial membrane 20 homolog (yeast) | -0.87 | -0.22 | 0.65 | 0 |
|  | Ipo11 | riken cdna 1700081h05 gene | 1.06 | 0.21 | -0.85 | 1 |
|  | Kpna3 | karyopherin (importin) alpha 3 | 1.39 | 0.07 | -1.33 | 0 |
|  | Kpnb1 | karyopherin (importin) beta 1 | 1.09 | 0.16 | -0.92 | 0 |
|  | Cd36 | cd36 antigen | 2.7 | 0.91 | -1.79 | 1 |
|  | Fmr1 | fragile x mental retardation syndrome 1 homolog | 1.67 | 0.15 | -1.52 | 0 |
|  | Itpr2 | inositol 1,4,5-triphosphate receptor 2 | 0.98 | 0.03 | -0.96 | 0 |
|  | Ramp2 | receptor (calcitonin) activity modifying protein 2 | 2.18 | 0.17 | -2.01 | 1 |
|  | Slc2a4 | solute carrier family 2 (facilitated glucose transporter), member 4 | -1.63 | -0.59 | 1.03 | 0 |
|  |  |  |  |  |  |  |
| **Transcription: factors, regulators** | |  |  |  |  |  |
|  | Cebpd | ccaat/enhancer binding protein (c/ebp), delta | -1.27 | -0.36 | 0.91 | 0 |
|  | Dr1 | down-regulator of transcription 1 | 1.1 | 0.27 | -0.83 | 0 |
|  | Hif1a | hypoxia inducible factor 1, alpha subunit | 1.17 | 0.03 | -1.14 | 0 |
|  | Hipk3 | homeodomain interacting protein kinase 3 | 1.18 | 0.05 | -1.13 | 1 |
|  | Nfatc3 | nuclear factor of activated t-cells, cytoplasmic, calcineurin-dependent 3 | 1.6 | 0.55 | -1.05 | 1 |
|  | Nr3c1 | nuclear receptor subfamily 3, group c, member 1 | 1.36 | 0.24 | -1.12 | 0 |
|  | Phr1 | pam, highwire, rpm 1 | 2.27 | 0.5 | -1.77 | 1 |
|  | Polr2j | polymerase (rna) ii (dna directed) polypeptide j | -1.01 | -0.13 | 0.88 | 0 |
|  | Rbpsuh | recombining binding protein suppressor of hairless (drosophila) | 1.18 | 0.3 | -0.88 | 0 |
|  | Rnf2 | ring finger protein 2 | 1.26 | 0.21 | -1.05 | 0 |
|  | Rnf141 | ring finger protein 141 | 1.19 | 0.14 | -1.05 | 0 |
|  | Rxrb | retinoid x receptor beta | -0.97 | -0.2 | 0.78 | 0 |
|  | Smarca2 | swi/snf related, matrix associated, actin dependent regulator of chromatin, subfamily a, member 2 | 1.01 | 0.09 | -0.91 | 0 |
|  | Tcea1 | transcription elongation factor a (sii) 1 | 1.3 | 0.14 | -1.16 | 0 |
|  | Tgif2 | tgfb-induced factor 2 | -0.84 | -0.17 | 0.67 | 0 |
|  | Wwtr1 | ww domain containing transcription regulator 1 | 1.76 | 0.38 | -1.38 | 0 |
|  | Zhx1 | zinc fingers and homeoboxes protein 1 | 1.8 | 0.45 | -1.35 | 0 |
|  | Rp9h | retinitis pigmentosa 9 homolog (human) | -1.24 | -0.29 | 0.94 | 0 |
|  | Creb3l2 | cAMP responsive element binding protein 3-like 2 | 1.02 | 0.32 | -0.70 | 0 |
|  | Cnot1 | CCR4-NOT transcription complex, subunit 1 | 0.86 | 0.10 | -0.76 | 0 |
|  | Fem1a | feminization 1 homolog a (C. elegans) | -1.09 | -0.01 | 1.08 | 0 |
|  | Copeb | Kruppel-like factor 6 | 1.21 | 0.09 | -1.12 | 0 |
|  | Lrrfip1 | leucine rich repeat (in FLII) interacting protein 1 | 1.42 | 0.44 | -0.98 | 0 |
|  | Lmo1 | LIM domain only 1 | 0.70 | 0.03 | -0.67 | 0 |
|  | Ell2 | Elongation factor RNA polymerase II 2 | 1.57 | 0.29 | -1.28 | 0 |
|  |  |  |  |  |  |  |
| **Nucleosome / Chromatin** | |  |  |  |  |  |
|  | Smarca2 | swi/snf related, matrix associated, actin dependent regulator of chromatin, subfamily a, member 2 | 1.01 | 0.09 | -0.91 | 0 |
|  | Nap1l1 | nucleosome assembly protein-1 | 1.24 | 0.19 | -1.05 | 0 |
|  | Chd9 | chromodomain helicase DNA binding protein 9 | 1.04 | 0.12 | -0.92 | 0 |
|  | Ncl | nucleolin | 1.70 | 0.09 | -1.62 | 0 |
|  |  |  |  |  |  |  |
| **Modification of DNA / RNA** | |  |  |  |  |  |
|  | Alkbh | alkB, alkylation repair homolog 1 (E. coli) | -1.02 | -0.25 | 0.77 | 0 |
|  | Rod1 | ROD1 regulator of differentiation 1 (S. pombe) | 1.75 | 0.37 | -1.38 | 0 |
|  | Fnbp3 | Formin binding protein 3 (Prpf40a) | 1.15 | 0.17 | -0.98 | 0 |
|  | Mbnl1 | Muscleblind-like 1 | 1.47 | 0.15 | -1.32 | 0 |
|  | Mbnl2 | Muscleblind-like 2 | 1.40 | -0.23 | -1.63 | 0 |
|  | AU067695 (Tsen2) | tRNA splicing endonuclease 2 homolog (SEN2, S. cerevisiae) | -0.92 | -0.17 | 0.74 | 1 |
|  | Pold2 | Polymerase (DNA directed), delta 2, regulatory subunit | -0.99 | -0.26 | 0.73 | 1 |
|  | Rbms1 | RNA binding motif, single stranded interacting protein 1 | 1.07 | 0.00 | -1.07 | 0 |
|  |  |  |  |  |  |  |
|  |  |  |  |  |  |  |
| **Cell proliferation / Cell growth / Cell differentiation / Apoptosis** | | | |  |  |  |
| cell proliferation | Ccng1 | cyclin g1 | 1.73 | 0.07 | -1.66 | 0 |
|  | Ppp1cb | protein phosphatase 1, catalytic subunit, beta isoform | 1.5 | 0.22 | -1.29 | 0 |
|  | Ppp3ca | Protein phosphatase 3, catalytic subunit, alpha isoform | 1.23 | 0.17 | -1.06 | 0 |
|  | Ptp4a1 | Protein tyrosine phosphatase 4a1 | 1.45 | 0.30 | -1.15 | 0 |
|  | sep.02 | septin 2 | 1.37 | 0.23 | -1.14 | 0 |
|  | sep.06 | septin 6 | -1.23 | -0.49 | 0.74 | 0 |
|  | Cdkl2 | cyclin-dependent kinase-like 2 (CDC2-related kinase) | 1.08 | 0.03 | -1.05 | 0 |
|  | Hrpt2 | Hyperparathyroidism 2 homolog (human) (Cdc73) | 1.20 | 0.37 | -0.83 | 0 |
|  | Nek7 | NIMA (never in mitosis gene a)-related kinase 7 | 1.45 | -0.01 | -1.46 | 1 |
|  | Sipa1l1 | signal-induced proliferation-associated 1 like 1 | 2.64 | 0.52 | -2.13 | 1 |
|  | Braf | Braf transforming gene | 0.99 | 0.23 | -0.76 | 0 |
|  | Calm1 | calmodulin 1 | 1.10 | 0.30 | -0.80 | 1 |
|  | Ddx3x | Fibroblast growth factor inducible 14 (DEAD/H (Asp-Glu-Ala-Asp/His) box polypeptide 3, X-linked) | 1.67 | -0.03 | -1.70 | 0 |
| cell growth | Mest | mesoderm specific gene | 4.49 | 2.60 | -1.89 | 1 |
|  | Igf1 | Insulin-like growth factor 1 | 1.73 | 0.68 | -1.05 | 0 |
|  | Igfbp5 | Insulin-like growth factor-binding protein 5 | 1.44 | 0.47 | -0.97 | 1 |
|  | Plac8 | Placenta-specific 8 | -0.85 | -0.22 | 0.63 | 0 |
| differentiation | Efna5 | ephrin a5 | 1.27 | 0.42 | -0.85 | 0 |
|  | Rfng | radical fringe gene homolog (drosophila) | -1.09 | -0.04 | 1.05 | 1 |
|  | Sema3c | sema domain, immunoglobulin domain (ig), short basic domain, secreted, (semaphorin) 3c | 1.77 | 0.06 | -1.71 | 0 |
|  | Gmfb | Glia maturation factor, beta | 1.33 | 0.02 | -1.31 | 0 |
|  | Fgf1 | fibroblast growth factor 1 | 0.87 | 0.12 | -0.75 | 1 |
|  | Itgav | integrin alpha v | 1.13 | 0.44 | -0.69 | 0 |
|  | Rnf6 | ring finger protein (c3h2c3 type) 6 | 1 | 0.03 | -0.96 | 0 |
|  | Wwtr1 | ww domain containing transcription regulator 1 | 1.76 | 0.38 | -1.38 | 0 |
|  | Fndc3b (1600019O04Rik) | fibronectin type III domain containing 3B | 1.69 | -0.15 | -1.85 | 0 |
| Apoptosis | Casp12 | caspase 12 | 1.42 | 0.48 | -0.94 | 0 |
|  | Ccar1 | cell division cycle and apoptosis regulator 1 | 1.47 | 0.31 | -1.16 | 0 |
|  |  |  |  |  |  |  |
| **Protein biosynthesis / Modification / Degradation** | | |  |  |  |  |
| biosynthesis | Bzw1 | basic leucine zipper and w2 domains 1 | 1.18 | -0.1 | -1.28 | 0 |
|  | Eif4g2 | eukaryotic translation initiation factor 4, gamma 2 | 1.71 | 0.3 | -1.41 | 0 |
|  | Eif5 | eukaryotic translation initiation factor 5 | 1.43 | 0.23 | -1.2 | 0 |
|  | Rpl18 | ribosomal protein l18 | -0.99 | -0.31 | 0.68 | 0 |
|  | Rpl28 | ribosomal protein l28 | -0.91 | -0.16 | 0.75 | 1 |
|  | Rpl41 | ribosomal protein l41 | -0.93 | -0.11 | 0.82 | 0 |
|  | Rps8 | ribosomal protein s8 | -1.1 | -0.36 | 0.74 | 1 |
|  | Rps10 | ribosomal protein s10 | -1.09 | -0.38 | 0.71 | 0 |
|  | Rps15 | ribosomal protein s15 | -0.9 | -0.13 | 0.78 | 0 |
|  | Transcribed locus, strongly similar to XP_344536.1 PREDICTED: similar to 40S ribosomal protein S28 [Rattus norvegicus] | | -0.85 | -0.05 | 0.80 | 1 |
|  | D3Jfr1 | Csde1: cold shock domain containing E1, RNA binding | 1.90 | -0.07 | -1.96 | 0 |
| Post-translational modification | C1galt1 | core 1 udp-galactose:n-acetylgalactosamine-alpha-r beta 1,3-galactosyltransferase | 0.87 | 0.06 | -0.81 | 0 |
|  | Pmm1 | Phosphomannomutase 1 | -1.65 | -0.58 | 1.07 | 0 |
|  | Siat10 | ST3 beta-galactoside alpha-2,3-sialyltransferase 6 (St3gal6) | 1.34 | 0.48 | -0.86 | 0 |
|  | Zdhhc2 | zinc finger, dhhc domain containing 2 | 1.89 | 0.83 | -1.06 | 1 |
|  | Zdhhc7 | zinc finger, dhhc domain containing 7 | -1.07 | -0.12 | 0.95 | 0 |
| Ubiquitin pathway / degradation | Cul1 | cullin 1 | 1.21 | 0.18 | -1.03 | 0 |
|  | Cul3 | cullin 3 | 1.56 | -0.02 | -1.58 | 0 |
|  | Fbxl5 | f-box and leucine-rich repeat protein 5 | 0.79 | 0.05 | -0.74 | 0 |
|  | Fbxw7 | f-box and wd-40 domain protein 7, archipelago homolog (drosophila) | -0.75 | -0.04 | 0.71 | 0 |
|  | Nedd4 | neural precursor cell expressed, developmentally down-regulted gene 4 | 1.27 | 0.29 | -0.97 | 0 |
|  | Phr1 | pam, highwire, rpm 1 | 2.27 | 0.5 | -1.77 | 1 |
|  | Rnf19 | ring finger protein (c3hc4 type) 19 | 1.34 | 0.35 | -0.99 | 0 |
|  | Usp46 | ubiquitin specific peptidase 46 | 1.12 | 0.2 | -0.91 | 0 |
|  | Wwp1 | ww domain containing e3 ubiquitin protein ligase 1 | 1.61 | 0.14 | -1.46 | 0 |
|  | Wsb1 | WD repeat and SOCS box-containing 1 | 0.97 | 0.11 | -0.86 | 0 |
|  | Znrf2 | zinc and ring finger 2 (D6Ertd365e) | 1.05 | -0.22 | -1.27 | 0 |
|  | Usp48 | ubiquitin specific peptidase 48 | 0.98 | 0.24 | -0.74 | 0 |
|  | Capn7 | calpain 7 | 1.56 | 0.32 | -1.24 | 0 |
|  | Dpp4 | dipeptidylpeptidase 4 | 1.40 | 0.08 | -1.32 | 0 |
|  | Edem1 | ER degradation enhancer, mannosidase alpha-like 1 | 1.32 | 0.24 | -1.08 | 0 |
|  | D19Wsu12e | DNA segment, Chr 19, Wayne State University 12, expressed | 1.18 | 0.35 | -0.83 | 0 |
|  | Pcyox1 | Prenylcysteine oxidase 1 | 0.95 | 0.31 | -0.64 | 1 |
|  | Serpinb6a | Receptor (TNFRSF)-interacting serine-threonine kinase 1 | 1.01 | 0.23 | -0.78 | 1 |
|  |  |  |  |  |  |  |
|  |  |  |  |  |  |  |
| **Role in Mitochondria / Oxidative phosphorylation** | | |  |  |  |  |
|  | Atp5k | atp synthase, h+ transporting, mitochondrial f1f0 complex, subunit e | -1.25 | 0.15 | 1.39 | 0 |
|  | Ndufb9 | nadh dehydrogenase (ubiquinone) 1 beta subcomplex, 9 | -1.2 | -0.16 | 1.03 | 1 |
|  | Ndufs8 | nadh dehydrogenase (ubiquinone) fe-s protein 8 | -0.94 | -0.05 | 0.89 | 0 |
|  | Np15 | Ndufb11:NADH dehydrogenase (ubiquinone) 1 beta subcomplex, 11 | -0.99 | 0.02 | 1.00 | 0 |
|  | Cyp4v3 | cytochrome P450, family 4, subfamily v, polypeptide 3 | 1.58 | 0.63 | -0.95 | 0 |
|  | Opa1 | optic atrophy 1 homolog (human) | 1.01 | 0.04 | -0.97 | 0 |
|  |  |  |  |  |  |  |
|  |  |  |  |  |  |  |
| **Lipid metabolism** | |  |  |  |  |  |
|  | Pcx | pyruvate carboxylase | -1.81 | -0.66 | 1.15 | 0 |
|  | Ggps1 | geranylgeranyl diphosphate synthase 1 | 1.03 | -0.06 | -1.09 | 0 |
|  | Large | like-glycosyltransferase | 1.02 | 0.41 | -0.61 | 1 |
|  | Fasn | fatty acid synthase | -4.52 | -2.86 | 1.66 | 0 |
|  | Scd2 | stearoyl-coenzyme a desaturase 2 | -1.11 | -1.9 | -0.79 | 1 |
|  | Ptges2 | prostaglandin e synthase 2 | -1.8 | -0.67 | 1.13 | 0 |
|  | Mlstd2 | male sterility domain containing 2 | 1.4 | -0.03 | -1.43 | 0 |
|  | Acadm | acetyl-coenzyme a dehydrogenase, medium chain | 1.64 | 0.64 | -1 | 1 |
|  | Vldlr | very low density lipoprotein receptor | 1.97 | 0.8 | -1.17 | 0 |
|  | Cav1 | caveolin, caveolae protein 1 | 2.05 | 0.62 | -1.43 | 1 |
|  | Osbpl8 | oxysterol binding protein-like 8 | 1.38 | 0.2 | -1.18 | 1 |
|  |  |  |  |  |  |  |
| **Metabolism** |  |  |  |  |  |  |
|  | Acbd5 | acyl-Coenzyme A binding domain containing 5 | 1.67 | 0.17 | -1.49 | 0 |
|  | Pyy | Peptide YY | 2.08 | 0.49 | -1.59 | 0 |
|  | Prps1 | Phosphoribosyl pyrophosphate synthetase 1 | 1.78 | 0.88 | -0.90 | 1 |
|  | Gls | Glutaminase | 1.73 | 0.00 | -1.74 | 0 |
|  |  |  |  |  |  |  |
|  |  |  |  |  |  |  |
| **Signaling / regulation of signal** | |  |  |  |  |  |
|  | Rgs11 | regulator of G-protein signaling 11 | 2.30 | 0.83 | -1.47 | 0 |
|  | Arhgef12 | Rho guanine nucleotide exchange factor (GEF) 12 | 1.05 | 0.26 | -0.79 | 1 |
|  | Wdr26 | WD repeat domain 26 | 1.14 | 0.06 | -1.08 | 0 |
|  | Ccpg1 | cell cycle progression 8 protein | 1.41 | 0.29 | -1.12 | 0 |
|  | Grb14 | Growth factor receptor-bound protein 14 | 2.72 | 1.76 | -0.96 | 1 |
|  | Mir16 | membrane interacting protein of RGS16 | -1.11 | -0.11 | 1.00 | 0 |
|  | Strn4 | striatin, calmodulin binding protein 4 | -1.04 | -0.46 | 0.58 | 0 |
|  | Pik3ca | phosphatidylinositol 3-kinase, catalytic, alpha polypeptide | 1.43 | 0.11 | -1.32 | 1 |
|  | AA960558=Plekha1 | Pleckstrin homology domain containing, family A (phosphoinositide binding specific) member 1 | 0.84 | 0.03 | -0.81 | 0 |
|  | Phip | Pleckstrin homology domain interacting protein | 1.32 | 0.14 | -1.18 | 0 |
|  | Rras2 | Related RAS viral (r-ras) oncogene homolog 2 | 0.84 | 0.06 | -0.78 | 0 |
|  | Prkcn | Protein kinase C, nu | 1.58 | -0.07 | -1.64 | 1 |
|  | Ryk | Receptor-like tyrosine kinase | 0.82 | 0.02 | -0.79 | 1 |
|  |  |  |  |  |  |  |
|  |  |  |  |  |  |  |
| **Others / Unknown function** | |  |  |  |  |  |
|  | Npr3 | natriuretic peptide receptor 3 (B430320C24Rik) | 4.07 | 1.25 | -2.82 | 1 |
|  | Rnf185 | ring finger protein 185 (1700022N24Rik) | -0.73 | -0.16 | 0.57 | 0 |
|  | Sh3bgrl | SH3-binding domain glutamic acid-rich protein like | 2.20 | 0.29 | -1.91 | 0 |
|  | Scoc | short coiled-coil protein | 1.49 | -0.16 | -1.65 | 0 |
|  | Bxdc1 | brix domain containing 1 | -0.74 | -0.16 | 0.58 | 0 |
|  | Cab39l | calcium binding protein 39-like | 1.11 | 0.26 | -0.86 | 0 |
|  | Arpp19 | cAMP-regulated phosphoprotein 19 | 1.59 | 0.08 | -1.52 | 0 |
|  | BC003331 | cDNA sequence BC003331 | 1.84 | 0.15 | -1.68 | 0 |
|  | BC031748 | cDNA sequence BC031748 | 1.45 | 0.22 | -1.23 | 0 |
|  | Ddx27 | DEAD (Asp-Glu-Ala-Asp) box polypeptide 27 | -0.99 | -0.35 | 0.64 | 0 |
|  | Klhl | kelch-like 9 (Drosophila) | 1.16 | 0.13 | -1.03 | 0 |
|  | Mospd1 | motile sperm domain containing 1 | 1.35 | 0.19 | -1.16 | 0 |
|  | Tm4sf9 | Tetraspanin 5 | -0.75 | -0.22 | 0.53 | 0 |
|  | F830029L24Rik | March6=Membrane-associated ring finger (C3HC4) 6 | 0.99 | 0.18 | -0.81 | 0 |
